# Predictive validity of the prognosis on admission aneurysmal subarachnoid haemorrhage scale for the outcome of patients with aneurysmal subarachnoid haemorrhage

**DOI:** 10.1038/s41598-023-33798-5

**Published:** 2023-04-25

**Authors:** Tuan Anh Nguyen, Luu Dang Vu, Ton Duy Mai, Co Xuan Dao, Hung Manh Ngo, Hai Bui Hoang, Son Ngoc Do, Hao The Nguyen, Dung Thi Pham, My Ha Nguyen, Duong Ngoc Nguyen, Hien Thi Thu Vuong, Hung Dinh Vu, Dong Duc Nguyen, Linh Quoc Nguyen, Phuong Viet Dao, Thanh Dang Vu, Dung Tien Nguyen, Tuan Anh Tran, Trang Quynh Pham, Chi Van Nguyen, Anh Dat Nguyen, Chinh Quoc Luong

**Affiliations:** 1 Center for Emergency Medicine, Bach Mai Hospital, 78 Giai Phong Road, Phuong Mai Ward, Dong Da District, Hanoi, 100000 Vietnam; 2 Department of Emergency and Critical Care Medicine, Hanoi Medical University, Hanoi, Vietnam; 3 Radiology Centre, Bach Mai Hospital, Hanoi, Vietnam; 4 Department of Radiology, Hanoi Medical University, Hanoi, Vietnam; 5 Stroke Center, Bach Mai Hospital, Hanoi, Vietnam; 6 Faculty of Medicine, University of Medicine and Pharmacy, Vietnam National University, Hanoi, Vietnam; 7 Center for Critical Care Medicine, Bach Mai Hospital, Hanoi, Vietnam; 8 Department of Neurosurgery II, Neurosurgery Center, Vietnam-Germany Friendship Hospital, Hanoi, Vietnam; 9 Department of Surgery, Hanoi Medical University, Hanoi, Vietnam; 10 Emergency and Critical Care Department, Hanoi Medical University Hospital, Hanoi Medical University, Hanoi, Vietnam; 11 Department of Neurosurgery, Bach Mai Hospital, Hanoi, Vietnam; 12 Department of Nutrition and Food Safety, Faculty of Public Health, Thai Binh University of Medicine and Pharmacy, Thai Binh, Vietnam; 13 Department of Health Organization and Management, Faculty of Public Health, Thai Binh University of Medicine and Pharmacy, Thai Binh, Vietnam; 14 Emergency Department, Vietnam-Czechoslovakia Friendship Hospital, Hai Phong, Vietnam; 15 Emergency Department, Agriculture General Hospital, Hanoi, Vietnam

**Keywords:** Cerebrovascular disorders, Headache, Hydrocephalus, Neurovascular disorders, Stroke, Risk factors, Cardiovascular diseases, Neurological disorders

## Abstract

This multicentre prospective cohort study aimed to compare the accuracy of the PAASH, WFNS, and Hunt and Hess (H&H) scales in predicting the outcomes of adult patients with aneurysmal SAH presented to three central hospitals in Hanoi, Vietnam, from August 2019 to June 2021. Of 415 eligible patients, 32.0% had a 90-day poor outcome, defined as an mRS score of 4 (moderately severe disability) to 6 (death). The PAASH, WFNS and H&H scales all have good discriminatory abilities for predicting the 90-day poor outcome. There were significant differences in the 90-day mean mRS scores between grades I and II (*p* = 0.001) and grades II and III (*p* = 0.001) of the PAASH scale, between grades IV and V (*p* = 0.026) of the WFNS scale, and between grades IV and V (*p* < 0.001) of the H&H scale. In contrast to a WFNS grade of IV–V and an H&H grade of IV–V, a PAASH grade of III–V was an independent predictor of the 90-day poor outcome. Because of the more clearly significant difference between the outcomes of the adjacent grades and the more strong effect size for predicting poor outcomes, the PAASH scale was preferable to the WFNS and H&H scales.

## Introduction

Subarachnoid haemorrhage (SAH) is a severe occurrence that is generally linked with high mortality and morbidity rates as well as a significant strain on health care^1,2^. In population-based studies, the death rate is around 50%, with a tendency toward steady improvement^3–5^. This fatality rate includes 10–18% of all aneurysmal SAH patients who die at home or while being transported to the hospital^6,7^. The main consequences of aneurysmal SAH related to initial bleeding, rebleeding, delayed cerebral ischaemia (DCI), hydrocephalus, elevated intracranial pressure (ICP), seizures, and cardiac problems can cause later early mortality among patients who arrive at the hospital alive^8–10^. Moreover, as compared to the general population, individuals with aneurysmal SAH who are discharged alive from the hospital have a higher long-term death rate^11–15^. Individuals who are alive when they are released from the hospital have a high risk of memory and neurocognitive impairment^16,17^. Global impairment was evident 3 months following aneurysmal clipping in roughly 20% of all patients who were released alive from the hospital and in 16% of those with favourable preoperative status^18^.

The degree of neurologic impairment and the extent of subarachnoid bleeding at the time of admission are the most important predictors of neurologic complications and outcomes^16,19^. Therefore, it is imperative to grade the severity of SAH as soon as feasible after the presentation and stabilization of patients with SAH. A number of grading systems are used in clinical practice to standardize the classification of patients with SAH based upon the initial evaluation.

The grading systems proposed by Hunt and Hess (H&H) and the World Federation of Neurological Surgeons (WFNS) are among the most widely used^20,21^. Although the H&H scale is easy to administer, the classifications are arbitrary, some of the terms are vague, and some patients may present with initial features that defy placement within a single grade^22^. As a result, the interobserver agreement for the H&H scale is poor^23,24^. A systematic review of SAH grading scales also found conflicting data regarding the utility of the H&H scale for prognosis^22^. Furthermore, it is unclear whether there are significant differences in outcomes for adjacent H&H grades^25–28^.

Unlike the H&H scale, the WFNS scale uses objective terminology to assign grades^22^. However, it may be more complex to administer than the H&H scale because it requires assessment of both the motor function and the Glasgow coma scale (GCS). One study of 50 patients with SAH found that the interobserver variability for the WFNS scale was moderate^24^. Additionally, a systematic review of SAH grading scales also found conflicting data regarding the prognostic power of the WFNS grades^22^. Therefore, making accurate initial predictions of the outcome after SAH remains a challenge.

Another 5-category grading scale, the Prognosis on Admission of Aneurysmal Subarachnoid Haemorrhage (PAASH) grading scale, has been developed based solely on the GCS^29^, which has a much better interobserver agreement^24^. A previous study showed that the PAASH scale had a good discriminatory ability for the prognosis of patients with aneurysmal SAH and was slightly preferable to the WFNS scale^30^. However, limited data on this scale’s external validity are available. The aim of this study was to investigate the rate of poor outcomes of patients with aneurysmal SAH, to determine the relationships among the grades on the PAASH, WFNS, and H&H scales and the actual outcomes and to compare the prognostic accuracy of these scales.

## Methods

### Source of data

This multicentre prospective observational study is the major update of our published previous study^31^, which collected data on patients with aneurysmal SAH consecutively admitted to the three national tertiary hospitals (Vietnam-Germany Friendship, Bach Mai, and Hanoi Medical University Hospital) in Hanoi, Vietnam, between August 2019 and June 2021. These hospitals are designated central hospitals in northern Vietnam by the Ministry of Health of Vietnam, of which the first is a surgical hospital with 1500 beds, the second is a large general hospital with 3200 beds, and the last is a small general hospital with 580 beds. Each participating hospital had at least two representatives (i.e., fully trained clinicians or surgeons) who were a part of the study team. Participation was voluntary and unfunded. All patients received a follow-up till discharge from the hospital or death in the hospital and had clinic visits or phone contacts on days 30th and 90th after ictus for the modified Rankin Scale (mRS) assessments, mRS ranges from 0 (no disability) to 6 (death)^32^, and evaluation of chronic hydrocephalus.

### Participants

This study included all patients (aged 18 years or older) presenting with aneurysmal SAH to the three central hospitals within 4 days of ictus. We defined a case of aneurysmal SAH as a person who had the presence of blood visible on a head computed tomography (CT) scan (or in case the CT scan was negative, the presence of xanthochromia in the cerebral spinal fluid) in combination with an aneurysm confirmed on CT or digital subtraction angiography (DSA)^16^. We excluded patients for whom the GCS on admission was unable to be scored (e.g., patients intubated and under sedation before arrival at the central hospital) or patients who became lost at 90 days of follow-up during the study period. In the case of aphasia, patients were classified according to the clinically possible GCS scores derived from their eye and motor scores^33,34^. When different possible verbal scores placed patients in different categories, these patients were excluded.

All patients were managed following the American Heart Association (AHA)/American Stroke Association (ASA) guidelines for the management of aneurysmal SAH^16^. Aneurysm repair with endovascular coiling or surgical clipping was performed as early as possible and immediately if rebleeding occurred. The decision to treat the cerebral aneurysms was made based on the discretion of the physician in charge of the patients and the availability of endovascular coiling or neurosurgical clipping, which depended on the participating hospital and the financial situation (either insurance or patient self-pay).

### Data collection

The data for each study patient were recorded from the same unified samples (case record form). A case record form (CRF) was adopted across the study sites to collect the common variables. Data were entered by a researcher or investigator into the study database via EpiData Entry software (EpiData Association, Denmark, Europe), which was used for simple or programmed data entry and data documentation that could prevent data entry errors or mistakes. We also checked the data for implausible outliers and missing fields and contacted hospital representatives for clarification. Patient identifiers were not entered into the database to protect the patients’ confidentiality.

### Outcome measures

The primary outcome of this study was poor neurological function (poor outcome) on day 90th after ictus, which was defined as mRS scores of 4 (moderately severe disability) to 6 (death)^35,36^. We also examined the following secondary outcomes: poor outcome on day 30th after ictus, 30- and 90-day mortality rates, and incidence rate of complications.

### Predictor measures

We defined exposure variables as SAH grading scales (i.e., the PAASH, WFNS, and H&H grading scales) at the time of admission to the hospital. Based on the admission GCS, we divided patients into the five categories of the PAASH grading scale, including grade I (GCS of 15), II (GCS of 11–14), III (GCS of 8–10), IV (GCS of 4–7), and V (GCS of 3)^29^, and into the five categories of the WFNS grading scale ranging from grade I (GCS score of 15) to V (GCS scores of 3–6), of which focal deficits make up 1 additional grade for patients with a GCS score of 14 or 13^21^. Based on the clinical condition on admission, we also classified patients into the five severity groups according to the H&H grading scale, which consists of five grades ranging from minimally symptomatic to coma^20^. All data elements required for calculating the GCS score and for classifying patients according to the PAASH, WFNS, or H&H grading scale at the time of admission to the hospital were prospectively assessed and collected on the same unified CRF by a fully trained clinician or surgeon of the participating hospitals and then were entered by a researcher or investigator into a study database via the EpiData Entry software for later analysis.

We determined confounding factors as variables collected on the same unified CRF by a fully trained clinician or surgeon. The CRF included variables based on the unruptured intracranial aneurysm (UIA) and SAH work group (WG) recommendations^37^, such as information on:

i. Medical histories (e.g., stroke, UIA, etc.) and clinical presentation (e.g., GCS and focal neurological signs).
ii. Admission head CT scan (e.g., presence of SAH, intraventricular haemorrhage (IVH) or intracerebral haemorrhage (ICH), and Fisher scale) and follow-up head CT scan during hospitalization (e.g., presence of SAH, IVH or ICH) or on days 30th and 90th after ictus (e.g., the presence of chronic hydrocephalus). We also collected data on the aneurysm site and aneurysm size from DSA or multi-slice CT (MSCT) angiography scan.
iii. Surgical and endovascular interventions (i.e., surgical clipping or endovascular coiling), rescue therapies (e.g., surgical haematoma evacuation, defined as any surgical procedure evacuating epidural, subdural, intraventricular, or intraparenchymal haematoma, such as decompressive craniectomy, open craniotomy, or minimally invasive surgery; external ventricular drain (EVD) placement; ventriculoperitoneal (VP) shunt), and intensive care unit (ICU) therapies (e.g., mechanical ventilation).
iv. Neurological complications (e.g., rebleeding, which included bleeding into the subarachnoid space, intracerebral, intraventricular, or subdural compartments; delayed cerebral ischaemia (DCI), hydrocephalus). Rebleeding from a ruptured aneurysm was classified into two subtypes: early or late rebleeding. We defined early or late rebleeding as rebleeding occurring in the hospital before or after an aneurysm repair, respectively.
v. Clinical time course (e.g., time from ictus to hospital arrival, length of hospitalization)
vi. We also collected data on demographics (i.e., sex, age) and system variables, which are available as the online supplement to a previously published paper^31^.

### Sample size

In the present study, poor neurological function on day 90th after the ictus served as the primary outcome. Therefore, based on the rate of poor neurological function on day 90th after the ictus (39.1%) reported in a previously published study^30^, we used the formula to find the minimum sample size for estimating a population proportion, with a confidence level of 95% and a confidence interval (margin of error) of ± 4.7%, and an assumed population proportion of 39.1%. As a result, our sample size should be at least 415 patients. Therefore, our sample size was sufficient and reflected a normal distribution.

$$n=\frac{{z}^{2}x \widehat{p}\left(1-\widehat{p}\right)}{{\varepsilon }^{2}}$$

where *z* is the *z* score (*z* score for a 95% confidence level is 1.96); *ε* is the margin of error (*ε* for a confidence interval of ± 4.7% is 0.047); $\hat{p}$ is the population proportion ($\hat{p}$ for a population proportion of 39.1% is 0.391); *n* is the sample size

### Statistical analyses

We used IBM® SPSS® Statistics 22.0 (IBM Corp., Armonk, United States of America) and Analyse-it statistical software (Analyse-it Software, Ltd., Leeds, United Kingdom) for data analysis. We report the data as numbers (no.) and percentages (%) for categorical variables and medians and interquartile ranges (IQRs) or means and standard deviations (SDs) for continuous variables. Furthermore, comparisons were made between poor and good outcomes at 30 and 90 days of ictus for each variable using the Chi-squared test or Fisher’s exact test for categorical variables and the Mann–Whitney U test, Kruskal–Wallis test, or one-way analysis of variance for continuous variables.

Odds ratios (ORs) for a poor outcome on days 30th and 90th after ictus with 95% confidence intervals (CIs) were calculated for each grade of the 5-category SAH grading scales (i.e., the PAASH, WFNS, and H&H scales) with a univariable logistic regression model, with grade I taken as the reference. In all of the SAH grading scales (i.e., the PAASH, WFNS, and H&H scales), significant intergrade differences in the outcome (mean mRS scores) on days 30th and 90th after ictus that were determined using the Kruskal–Wallis H test with the Dunn-Bonferroni principle as a post hoc analysis.

We converted from descriptive SAH grading scales (i.e., the PAASH, WFNS, and H&H scales) to numerical SAH grading scales in ascending order (Table S1 as shown in Additional file 1). Receiver operator characteristic (ROC) curves were plotted, and the areas under the ROC curve (AUROC) were calculated to determine the discriminatory ability of all SAH grading scales for the prognosis of the patients at the time of evaluation. The cut-off value of each SAH grading scale was determined by ROC curve analysis and defined as the cut-off point with the maximum value of Youden’s index (i.e., sensitivity + specificity − 1). Based on the cut-off value of the scales, we assigned the patients to two severity groups: either the grade that was less than the cut-off value or another that was greater than or equal to the cut-off value. We also performed a pairwise comparison among the AUROCs of the PAASH, WFNS, and H&H scales for predicting the poor outcome on days 30th and 90th after ictus by using the Z-statistics.

We assessed the factors associated with 90-day poor outcomes using logistic regression analysis. To reduce the number of predictors and the multicollinearity issue and resolve the overfitting, we used different methods to select variables as follows: (a) we put all variables (including exposure and confounding factors) of demographics, baseline characteristics, clinical and laboratory characteristics, neuroimaging findings, clinical time course, treatments, and complications into the univariable logistic regression analyses; (b) we selected variables if the *p* value was < 0.05 in the univariable logistic regression analyses between the good and poor outcomes on day 90th after ictus, as well as those that are clinically crucial to put in the multivariable logistic regression model. These variables included demographics (i.e., age), risk factors for aneurysmal SAH (i.e., hypertension), comorbidities (i.e., diabetes mellitus), initial neuroimaging findings (i.e., location of blood within the subarachnoid space, the occurrence of IVH and ICH, and aneurysm location), the severity of the aneurysmal SAH on admission (i.e., the grades of PAASH, WFNS, and H&H grading scales that was either greater than or equal to the cut-off value), treatments (i.e., aneurysm repairs, nimodipine), and complications (i.e., rebleeding, DCI, acute hydrocephalus, and pneumonia). Using a stepwise backward elimination method, we started with the full multivariable logistic regression model that included the selected variables. This method then deleted the variables stepwise from the full model until all remaining variables were independently associated with the risk of 90-day poor outcomes in the final model. Similarly, we used these methods of variable selection and analysis for assessing factors associated with 30-day poor outcomes. For examining the effect size of each grade of the SAH grading scales, in combination with confounding factors, for predicting the 30- and 90-day poor outcomes, we replaced the severity group variables (i.e., the grades of PAASH, WFNS, and H&H grading scales that was either greater than or equal to the cut-off value) with each SAH grading scale (i.e., the PAASH, WFNS, or H&H scale), with grade I taken as the reference, in this multivariable logistic regression model, with the same set of confounding variables. We presented the odds ratios (ORs) and 95% confidence intervals (CIs) in the univariable logistic regression analyses and the adjusted odds ratios (AORs) and 95% CIs in the multivariable logistic regression models.

For all analyses, the significance levels were two-tailed, and we considered *p* < 0.05 to be statistically significant.

### Ethical issues

The Hanoi Medical University (Approval number: 3335/QĐ-ĐHYHN), Vietnam-Germany Friendship Hospital (Approval number: 818/QĐ-VĐ; Research code: KH04.2020), and Bach Mai Hospital (Approval number: 3288/QĐ-BM; Research code: BM_2020_1247) Scientific and Ethics Committees approved this study. This study was conducted according to the principles of the Declaration of Helsinki. The Vietnam-Germany Friendship Hospital Scientific and Ethics Committees waived written informed consent for this non interventional study, and public notification of the study was made by public posting, according to the Transparent Reporting of a multivariable prediction model for Individual Prognosis or Diagnosis (TRIPOD) statement—the TRIPOD checklist—for reporting a study developing or validating a multivariable prediction model for diagnosis or prognosis^38^. The authors who performed the data analysis kept the datasets in password-protected systems, and we only present anonymized data.

## Results

**Figure 1:**
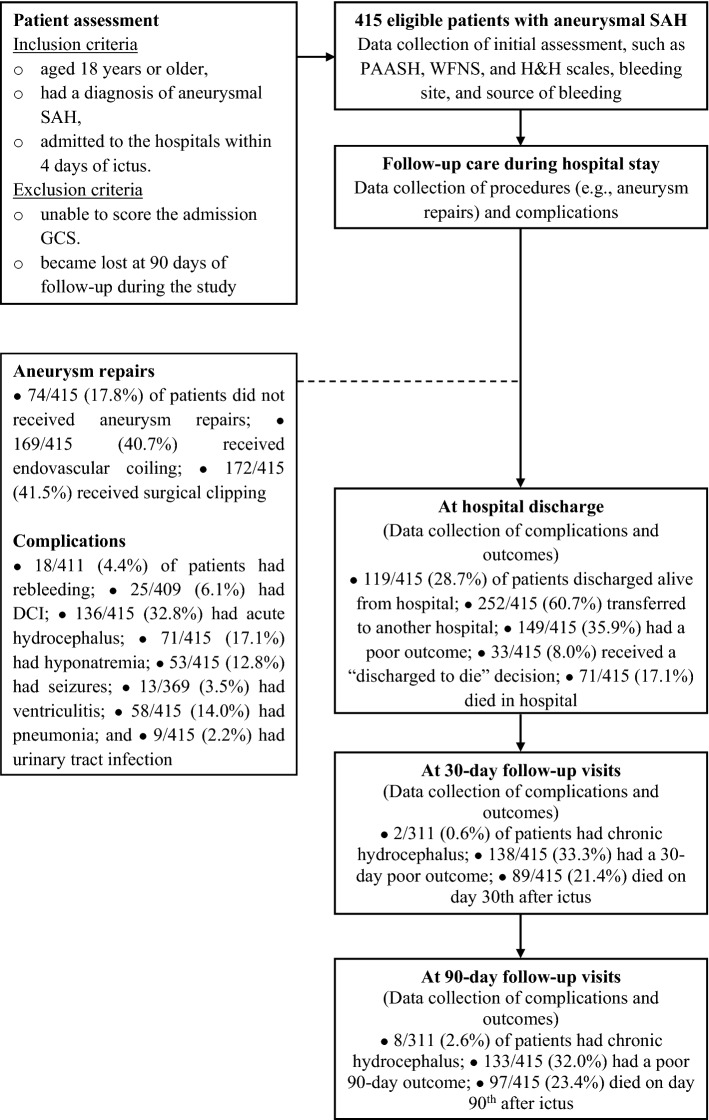
Flowchart of the study design and assessment occasions (“*discharged to die*”, defined as patients were in grave condition or dying and were classified with a modified Rankin Scale score of 5 (severe disability) at the time of discharge, *DCI* delayed cerebral ischaemia, *GCS* Glasgow coma scale, *H&H* Hunt and Hess grading scale, *PAASH* prognosis on admission aneurysmal subarachnoid haemorrhage grading scale, *poor outcome* defined as modified Rankin Scale scores of 4 (moderately severe disability) to 6 (death), *SAH* subarachnoid haemorrhage, *WFNS* World Federation of Neurosurgical Societies grading scale).

A total of 415 eligible patients presented to the study sites with aneurysmal SAH during the observation period (Fig. 1 and Table 1), data on these patients were entered into the study database by researchers or investigators, and there were few missing data.

**Table 1:** Baseline characteristics, treatments, complications, and outcomes of patients with aneurysmal subarachnoid haemorrhage according to neurologic function on day 90th after ictus.

	All cases	mRS of 0 to 3	mRS of 4 to 6	*p* value^a^
Demographics	n = 415	n = 282	n = 133	
Age (year), median (IQR)	57.0 (48.0–67.0)	56.0 (46.0–64.0)	64.0 (53.0–72.5)	< 0.001***
Gender (male), no. (%)	198 (47.7)	134 (47.5)	64 (48.1)	0.909
Risk factors for aneurysmal SAH	n = 415	n = 282	n = 133	
Cigarette smoking, no. (%)	159 (38.3)	103 (36.5)	56 (42.1)	0.275
Hypertension, no. (%), n = 413	163 (39.5)	94 (33.6)	69 (51.9)	< 0.001
Alcohol consumption, no. (%), n = 401	182 (45.4)	119 (44.1)	63 (48.1)	0.449
Comorbidities	n = 415	n = 282	n = 133	
Cerebrovascular disease, no. (%)	18 (4.3)	10 (3.5)	8 (6.0)	0.249
Chronic cardiac failure, no. (%)	7 (1.7)	3 (1.1)	4 (3.0)	0.218*
COPD/Asthma, no. (%)	5 (1.2)	3 (1.1)	2 (1.5)	0.657*
Diabetes mellitus, no. (%)	27 (6.5)	10 (3.5)	17 (12.8)	< 0.001
Clinical presentation on admission	n = 415	n = 282	n = 133	
GCS score, median (IQR)	14 (10.0–15.0)	15 (14.0–15.0)	8 (6.0–12.0)	< 0.001**
Focal neurological deficits, no. (%)	315 (75.9)	202 (71.6)	113 (85.0)	0.003
Neuroimaging findings on admission	n = 415	n = 282	n = 133	
Blood filling the subarachnoid space, no. (%)				
Basal cistern, n = 411	228 (55.5)	135 (48.0)	93 (71.5)	< 0.001
Sylvian fissure, n = 413	380 (92.0)	253 (90.0)	127 (96.2)	0.031
Interhemispheric fissure, n = 412	291 (70.6)	186 (66.2)	105 (80.2)	0.004
Interpeduncular fossa, n = 412	266 (64.6)	160 (56.9)	106 (80.9)	< 0.001
Suprasellar cistern, n = 412	270 (65.5)	172 (61.2)	98 (74.8)	0.007
Ambient cistern, n = 412	258 (62.6)	155 (55.2)	103 (78.6)	< 0.001
Quadrigeminal cistern, n = 412	126 (30.6)	53 (18.9)	73 (55.7)	< 0.001
IVH, no. (%)	275 (66.3)	168 (59.6)	107 (80.5)	< 0.001
ICH, no. (%)	85 (20.5)	48 (17.0)	37 (27.8)	0.011
ICH volume (mL), mean (SD), n = 85	22.6 (22.82)	17.79 (16.88)	28.85 (27.78)	0.134
Aneurysm locations, no. (%)				
PCoA aneurysm	65 (15.7)	50 (17.7)	15 (11.3)	0.091
VA aneurysm	18 (4.3)	7 (2.5)	11 (8.3)	0.007
The admission severity	n = 415	n = 282	n = 133	
PAASH score^b^, median (IQR)	2.0 (1.0–3.0)	1.0 (1.0–2.0)	3.0 (2.0–4.0)	< 0.001**
WFNS score^b^, median (IQR)	2.0 (1.0–4.0)	1.0 (1.0–2.0)	4.0 (4.0–5.0)	< 0.001**
H&H score^b^, median (IQR)	2.0 (2.0–4.0)	2.0 (2.0–3.0)	5.0 (3.0–5.0)	< 0.001**
Aneurysm repairs and other treatments	n = 415	n = 282	n = 133	
Aneurysm repairs				
No aneurysm repair, no. (%)	74 (17.8)	5 (1.8)	69 (51.9)	< 0.001
Endovascular coiling, no. (%)	169 (40.7)	147 (52.1)	22 (16.5)	< 0.001
Surgical clipping, no. (%)	172 (41.5)	130 (46.1)	42 (31.6)	0.005
Surgical haematoma evacuation^c^, no. (%)	44 (10.6)	23 (8.2)	21 (15.8)	0.018
EVD, no. (%), n = 414	43 (10.4)	18 (6.4)	25 (18.8)	< 0.001
Nimodipine, no. (%), n = 166	331 (91.2)	239 (98.0)	92 (77.3)	< 0.001
Complications	n = 415	n = 282	n = 133	
Rebleeding, no. (%), n = 411	18 (4.4)	5 (1.8)	13 (10.0)	< 0.001
Early rebleeding, no. (%), n = 13	1 (7.7)	0 (0.0)	1 (8.3)	> 0.999
Late rebledding, no. (%), n = 13	12 (92.3)	1 (100)	11 (91.7)	> 0.999
DCI, no. (%), n = 409	25 (6.1)	7 (2.5)	18 (13.8)	< 0.001
Acute hydrocephalus, no. (%)	136 (32.8)	69 (24.5)	67 (50.4)	< 0.001
Pneumonia, no. (%)	58 (14.0)	23 (8.2)	35 (26.3)	< 0.001
Clinical time course	n = 415	n = 282	n = 133	
Ictus to hospital arrival (hour), no. (%), n = 408				0.089
≤ 24 h	212 (52.0)	134 (48.6)	78 (59.1)	
> 24–72 h	188 (46.0)	135 (48.9)	53 (40.1)	
> 72 h	8 (2.0)	7 (2.5)	1 (0.8)	
Length of hospitalization (days), mean (SD)	10.14 (9.85)	11.22 (9.5)	7.84 (10.22)	< 0.001**
Clinical outcomes	n = 415	n = 282	n = 133	
Deaths				
Died in hospital, no. (%)	71 (17.1)	0 (0.0)	71 (53.4)	< 0.001
Died on day30th after ictus, no. (%)	89 (21.4)	0 (0.0)	89 (66.9)	< 0.001
Died on day 90th after ictus, no. (%)	97 (23.4)	0 (0.0)	97 (72.9)	< 0.001
Neurological function				
mRS at hospital discharge, no. (%)				< 0.001
Good (mRS of 0–3)	266 (64.1)	262 (92.9)	4 (3.0)	
Poor (mRS of 4–6)	149 (35.9)	20 (7.1)	129 (97.0)	
mRS on day 30th after ictus, no. (%)				< 0.001
Good (mRS of 0–3)	277 (66.7)	277 (98.2)	0 (0.0)	
Poor (mRS of 4–6)	138 (33.3)	5 (1.8)	133 (100)	

See Table S2 to S9, as shown in Additional file 1, for additional information.

*COPD* chronic obstructive pulmonary disease, *DCI* delayed cerebral ischaemia, *EVD* external ventricular drainage, *GCS* Glasgow coma scale, *H&H* Hunt and Hess, *ICH* intracerebral haemorrhage, *IQR* interquartile range, *IVH* intraventricular haemorrhage, *mRS* modified Rankin Scale, *no.* number, *PAASH* Prognosis on Admission of Aneurysmal Subarachnoid Haemorrhage, *PCoA* posterior communicating artery, *SAH* subarachnoid haemorrhage, *SD* standard deviation, *VA* vertebral artery, *WFNS* World Federation of Neurosurgical Societies.

*Fisher's exact test.

**Mann–Whitney U test.

^a^Comparison between mRS of 0–3 and mRS of 4–6 using Chi-squared test.

^b^The descriptive SAH grading scales (i.e., the PAASH, WFNS, and H&H scales) were converted to the numerical SAH grading scales in ascending order (see Table S1, as shown in Additional file 1, for additional information).

^c^Surgical haematoma evacuation was defined as any surgical procedure evacuating epidural, subdural, intraventricular, or intraparenchymal haematoma, such as decompressive craniotomy, open craniotomy, or minimally invasive surgery.

### Baseline characteristics and clinical outcomes

Of the total patients, 198/415 (47.7%) were men, and the median age was 57.0 (IQR: 48.0–67.0) (Table 1). On admission, the median GCS score was 14 (IQR: 10.0–15.0), and focal neurological deficits were observed in 75.9% (315/415) of patients (Table 1). In addition, IVH and ICH were also detected on the admission CT scan in 66.3% (275/415) and 20.5% (85/415) of patients, respectively (Table 1). When we converted from the descriptive SAH grading scales to the numerical SAH grading scales in ascending order (Table S1 as shown in Additional file 1), the median PAASH score was 2.0 (IQR: 1.0–3.0), the median WFNS score was 2.0 (IQR: 1.0–4.0), and the median H&H score was 2.0 (IQR: 2.0–4.0) upon admission to the hospital (Table 1). Approximately two-fifths of the patients (40.7%; 169/415) were treated with endovascular coiling, 41.5% (172/415) were treated with surgical clipping, and the remaining patients (17.8%; 74/415) did not receive aneurysm repair (Table 1). Rebleeding and DCI occurred in 4.4% (18/411) and 6.1% (25/409) of patients, respectively (Table 1). Overall, 32.0% (133/415) of patients with aneurysmal SAH had a poor outcome on day 90th after ictus, 23.4% (97/415) of whom died within 90 days of ictus (Table 1). The characteristics, management, complications, and outcomes of the patients were compared between patients who had a good outcome and patients who had a poor outcome on days 30th and 90th after ictus, as shown in Table 1 and Tables S2 to S9 (Additional file 1).

### Overall prognostic performance of the SAH grading scales

**Figure 2:**
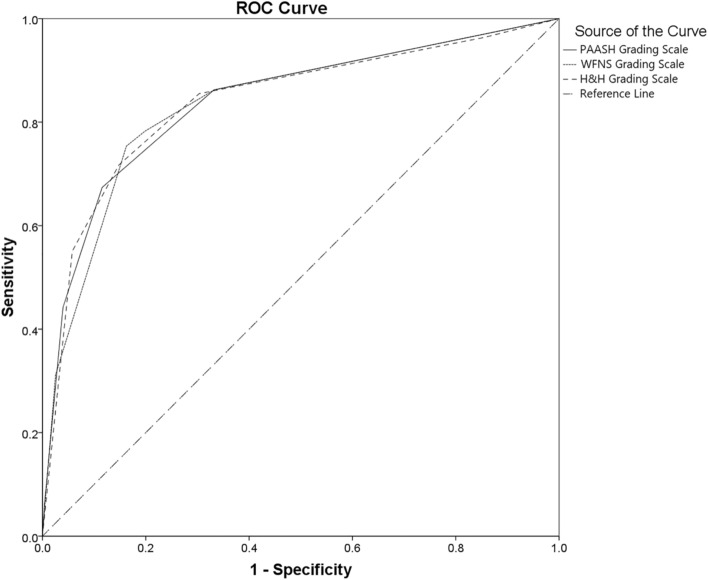
The overall prognostic performance of the SAH grading scales for the poor outcomes on day 30th after ictus: The area under the ROC curves of the PAASH (AUROC: 0.840 [95% CI 0.796–0.883]; cut-off value: ≥ 2.5; sensitivity: 67.4%; specificity: 88.4%; P_AUROC_ < 0.001), the WFNS (AUROC: 0.836 [95% CI 0.793–0.880]; cut-off value: ≥ 3.5; sensitivity: 75.4%; specificity: 83.8%; P_AUROC_ < 0.001), and the H&H scales (AUROC: 0.839 (95% CI 0.795–0.883); cut-off value: ≥ 3.5; sensitivity: 71.7%; specificity: 85.2%; P_AUROC_ < 0.001) for predicting the poor outcomes on day 30th after ictus in patients with aneurysmal SAH (*AUROC* areas under the receiver operating characteristic curve, *H&H* Hunt and Hess, *PAASH* prognosis on admission of aneurysmal subarachnoid haemorrhage, *poor outcome* defined as a modified Rankin Scale [mRS] score of 4–6, *ROC* receiver operating characteristic, *SAH* subarachnoid haemorrhage, *WFNS* World Federation of Neurological Surgeons).

**Figure 3:**
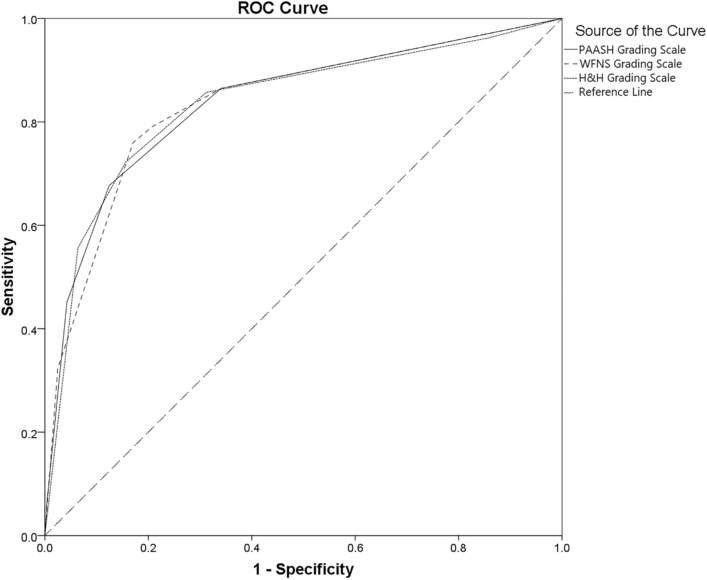
The overall prognostic performance of the SAH grading scales for the poor outcomes on day 90th after ictus: The area under the ROC curves of the PAASH (AUROC: 0.838 [95% CI 0.794–0.882]; cut-off value: ≥ 2.5; sensitivity: 67.7%; specificity: 87.6%; P_AUROC_ < 0.001), the WFNS (AUROC: 0.837 [95% CI 0.793–0.881]; cut-off value: ≥ 3.5; sensitivity: 75.9%; specificity: 83.0%; P_AUROC_ < 0.001), and the H&H scales (AUROC: 0.836 [95% CI 0.791–0.881]; cut-off value: ≥ 3.5; sensitivity: 72.2%; specificity: 84.4%; P_AUROC_ < 0.001) for predicting the poor outcomes on day 90th after ictus in patients with aneurysmal SAH (*AUROC* areas under the receiver operating characteristic curve, *H&H* Hunt and Hess, *PAASH* prognosis on admission of aneurysmal subarachnoid haemorrhage, *poor outcome* defined as a modified Rankin scale [mRS] score of 4–6, *ROC* receiver operating characteristic, *SAH* subarachnoid haemorrhage, *WFNS* World Federation of Neurological Surgeons).

Figures 2 and 3 show the overall performances of the SAH grading scales (i.e., the PAASH, WFNS, and H&H scales) for predicting the poor outcome, of which the PAASH (AUROC: 0.838 [95% CI 0.794–0.882]; cut-off value: ≥ 2.5; sensitivity: 67.7%; specificity: 87.6%; P_AUROC_ < 0.001), the WFNS (AUROC: 0.837 [95% CI 0.793–0.881]; cut-off value: ≥ 3.5; sensitivity: 75.9%; specificity: 83.0%; P_AUROC_ < 0.001), and the H&H scales (AUROC: 0.836 [95% CI 0.791–0.881]; cut-off value: ≥ 3.5; sensitivity: 72.2%; specificity: 84.4%; P_AUROC_ < 0.001) all had good discriminatory abilities for predicting the poor outcome on day 90th after ictus (Fig. 3). There were also the good discriminatory abilities of the SAH grading scales for predicting the poor outcome on day 30th after ictus, as shown in Fig. 2 and Table S10 (Additional file 1).

Table 2 shows the differences between the AUROC curves among different test-pairwise, of which we found that the AUROCs, which for predicting the poor outcome on day 90th after ictus, did not differ significantly among the PAASH and WFNS scales (AUROC difference: 0.001; 95% CI − 0.009 to 0.011; Z-statistic: 0.19; *p* = 0.849), the PAASH and H&H scales (AUROC difference: 0.002; 95% CI − 0.016 to 0.019; Z-statistic: 0.17; *p* = 0.862), and the WFNS and H&H scales (AUROC difference: 0.001; 95% CI − 0.017 to 0.018; Z-statistic: 0.07; *p* = 0.947). For predicting the poor outcome on day 30th after ictus, there were also no significant differences between the AUROC curves among different test-pairwise, as shown in Table 2.

**Table 2:** Pairwise comparisons of AUROC of the PAASH, WFNS, and H&H scales for predicting the poor outcome (mRS of 4 to 6) after the onset of haemorrhage in patients with aneurysmal SAH.

Comparisons	AUROC difference (95% CI)	SE	Z-statistic	*p* value
Poor outcome on day 30th after the onset of hemorrhage				
PAASH and WFNS	0.003 (− 0.006 to 0.013)	0.0059	0.55	0.584
PAASH and H&H	0.000 (− 0.017 to 0.018)	0.0104	0.04	0.968
WFNS and H&H	0.003 (− 0.014 to 0.020)	0.0104	0.27	0.788
Poor outcome on day 90th after the onset of hemorrhage				
PAASH and WFNS	0.001 (− 0.009 to 0.011)	0.0060	0.19	0.849
PAASH and H&H	0.002 (− 0.016 to 0.019)	0.0106	0.17	0.862
WFNS and H&H	0.001 (− 0.017 to 0.018)	0.0106	0.07	0.947

*AUC* the area under the curve, *AUROC* the area under the receiver operating characteristic, *H&H* Hunt and Hess scale, *PAASH* prognosis on admission of aneurysmal subarachnoid haemorrhage scale, *SAH* subarachnoid haemorrhage, *SE* standard error, *WFNS* World Federation of Neurosurgical Societies scale.

### Differences between the clinical outcomes of the adjacent grades

There were no significant differences in the 90-day mean mRS scores among all adjacent grades of the SAH grading scales (i.e., PAASH, WFNS, and H&H scales) except between grades I and II (0.63 ± 1.55 vs. 1.93 ± 2.50, respectively, *p* = 0.001) and grades II and III (1.93 ± 2.50 vs. 3.60 ± 2.50, respectively, *p* = 0.001) of the PAASH scale, between grades IV and V (3.75 ± 2.46 vs. 5.24 ± 1.68, respectively, *p* = 0.026) of the WFNS scale, and between grades IV and V (2.96 ± 2.60 vs. 4.97 ± 1.87, respectively, *p* < 0.001) of the H&H scale (Table 3). The difference in the 30-day mean mRS scores between the adjacent grades of the SAH grading scales (i.e., the PAASH, WFNS, and H&H scales) is available in Table 3.

**Table 3:** Comparison of outcomes between the intergrades of the subarachnoid haemorrhage grading scales.

SAH grading scale	Day 30th after ictus			Day 90th after ictus		
	mRS, mean (SD)	No. of patients	*p* value^a^	mRS, mean (SD)	No. of patients	*p* value^a^
PAASH scale						
I	0.69 (1.50)	204	NA	0.63 (1.55)	204	NA
II	2.12 (2.4)	86	< 0.001	1.93 (2.50)	86	0.001
III	3.75 (2.40)	53	0.003	3.60 (2.50)	53	0.001
IV	5.13 (1.67)	63	0.107	5.08 (1.79)	63	0.055
V	5.44 (1.67)	9	> 0.999	5.44 (1.67)	9	> 0.999
WFNS scale						
I	0.69 (1.50)	204	NA	0.63 (1.55)	204	NA
II	1.63 (2.29)	48	0.096	1.40 (2.35)	48	0.916
III	2.14 (2.41)	14	> 0.999	1.86 (2.51)	14	> 0.999
IV	3.86 (2.36)	99	0.403	3.75 (2.46)	99	0.159
V	5.26 (1.60)	50	0.051	5.24 (1.68)	50	0.026
H&H scale						
I	0.98 (1.79)	45	NA	0.87 (1.87)	45	NA
II	0.65 (1.45)^b^	168	> 0.999	0.59 (1.50)^b^	168	> 0.999
III	2.10 (2.44)	62	0.391	1.89 (2.49)	62	0.588
IV	3.06 (2.47)	48	0.306	2.96 (2.60)	48	0.211
V	5.03 (1.76)	92	0.001	4.97 (1.87)	92	< 0.001

*H&H* Hunt and Hess, *mRS* modified rankin scale, *NA* not applicable, *No.* number, *PAASH* prognosis on admission of aneurysmal subarachnoid haemorrhage, *SAH* subarachnoid haemorrhage, *SD* standard deviation, *WFNS* World Federation of Neurological Surgeons.

^a^Probability values were obtained by comparing the mean mRS score of a given grade with that of the mRS score just above it (nonparametric test by Dunn’s multiple comparisons).

^b^The grade of the H&H scale shows the reversed rank order of the mean mRS scores.

### Associations between the grading scales and clinical outcomes

In the univariable logistic regression analyses, we found that most grades of the SAH grading scales (i.e., PAASH, WFNS, and H&H scales) were significantly associated with the increased risk of poor outcome on days 30th and 90th after ictus, with grade I taken as a reference, except for associations between grade II of the H&H scale and the poor outcome on day 30th (OR: 0.727; 95% CI 0.247–2.139; *p* = 0.563) and day 90th (OR: 0.727; 95% CI 0.247–2.139; *p* = 0.563) after ictus (Table 4).

**Table 4:** OR for a poor outcome (mRS of 4 to 6) for the SAH grading scales.

SAH grading scale	Poor outcome on day 30th after ictus				Poor outcome on day 90th after ictus			
	N	mRS: 4–6, no. (%)	OR (95% CI)	*p* value	N	mRS: 4–6, no. (%)	OR (95% CI)	*p* value
PAASH scale								
I	204	19 (9.3)	Reference	< 0.001	204	18 (8.8)	Reference	< 0.001
II	86	26 (30.2)	4.219 (2.182–8.158)	< 0.001	86	25 (29.1)	4.235 (2.164–8.287)	< 0.001
III	53	32 (60.4)	14.837 (7.185–30.641)	< 0.001	53	30 (56.6)	13.478 (6.512–27.896)	< 0.001
IV	63	53 (84.1)	51.605 (22.630–117.682)	< 0.001	63	52 (82.5)	48.848 (21.716–109.879)	< 0.001
V	9	8 (88.9)	77.895 (9.240–656.657)	< 0.001	9	8 (88.9)	82.667 (9.781–698.704)	< 0.001
WFNS scale								
I	204	19 (9.3)	Reference	< 0.001	204	18 (8.8)	Reference	< 0.001
II	48	11 (22.9)	2.895 (1.272–6.587)	0.011	48	10 (20.8)	2.719 (1.164–6.350)	0.021
III	14	4 (28.6)	3.895 (1.114–13.621)	0.033	14	4 (28.6)	4.133 (1.177–14.520)	0.027
IV	99	61 (61.6)	15.630 (8.391–29.117)	< 0.001	99	58 (58.6)	14.618 (7.803–27.383)	< 0.001
V	50	43 (86.0)	59.812 (23.648–151.281)	< 0.001	50	43 (86.0)	63.476 (24.947–161.511)	< 0.001
H&H scale								
I	45	5 (11.1)	Reference	< 0.001	45	5 (11.1)	Reference	< 0.001
II	168	15 (8.9)	0.784 (0.269–2.287)	0.656	168	14 (8.3)	0.727 (0.247–2.139)	0.563
III	62	19 (30.6)	3.535 (1.206–10.358)	0.021	62	18 (29.0)	3.273 (1.112–9.631)	0.031
IV	48	23 (47.9)	7.360 (2.478–21.860)	< 0.001	48	22 (45.8)	6.769 (2.277–20.121)	0.001
V	92	76 (82.6)	38.000 (12.973–111.306)	< 0.001	92	74 (80.4)	32.889 (11.362–95.201)	< 0.001

*CI* confidence interval, *H&H* Hunt and Hess, *mRS* modified Rankin Scale, *N* total number of patients for each grade, *no.* number, *OR* odds ratio, *PAASH* Prognosis on Admission of Aneurysmal Subarachnoid Haemorrhage, *SAH* subarachnoid haemorrhage, *SD* standard deviation, *WFNS* World Federation of Neurological Surgeons.

When we added each SAH grading scale (i.e., PAASH, WFNS, or H&H scale), with grade I taken as the reference, to the multivariable logistic regression model, with the same set of confounding variables, for predicting the poor outcome on days 30th and 90th after ictus (Tables S11 to S16 as shown in Additional file 1), we also found that most grades of the PAASH, WFNS, and H&H scales were independently associated with the increased risk of poor outcome on day 90th after ictus, except for grade II (AOR: 3.112; 95% CI 0.970–9.977; *p* = 0.056) of the PAASH scale, grade II (AOR: 2.725; 95% CI 0.635–11.686; *p* = 0.177) and grade III (AOR: 4.813; 95% CI 0.691–33.541; *p* = 0.113) of the WFNS scale, and grade II (AOR: 3.596; 95% CI 0.596–21.685; *p* = 0.163) of the H&H scale (Tables S14 to S16, as shown in Additional file 1). Associations between the grades of the PAASH, WFNS, and H&H scales and the risk of the poor outcome on day 30th after ictus can be found in Tables S11 to S13, as shown in Additional file 1.

### Risk factors for the poor outcome

Tables 5 and 6 show the factors associated with the risk of poor outcomes. Based on the cut-off value of the PAASH (≥ 2.5), WFNS (≥ 3.5), or H&H scales (≥ 3.5) for predicting the poor outcome on days 30th and 90th after ictus (Figs. 2 and 3, Table S10 as shown in Additional file 1), the patients were classified into two groups: (1) either a PAASH grade of I–II or a grade of III–V, (2) either a WFNS grade of I–III or a grade of IV–V, or (3) either an H&H grade of I–III or a grade of IV–V. In the univariable logistic regression analyses, a PAASH grade of III–V (OR: 14.771; 95% CI 8.894–24.531; *p* < 0.001), a WFNS grade of IV–V (OR: 15.387; 95% CI 9.291–25.483; *p* < 0.001), and an H&H grade of IV–V (OR: 14.034; 95% CI 8.535–23.076; *p* < 0.001) were significantly associated with the increased risk of the poor outcome on day 90th after ictus (Table 6). However, the multivariate logistic regression model revealed that a PAASH grade of III–IV (AOR: 10.868; 95% CI 4.281–27.592; *p* < 0.001) was independently associated with the increased risk of the poor outcome on day 90th after ictus, in contrast to a WFNS grade of IV–V or an H&H grade of IV–V, for which this independent association was not found (Table 6). Although a PAASH grade of III–V, a WFNS grade of IV–V, and an H&H grade of IV–V were significantly associated with the increased risk of poor outcome on day 30th after ictus (Table 5), only a PAASH grade of III–V was an independent predictor of the poor outcome on day 30th after ictus, as shown in Table 5.

**Table 5:** Factors associated with 30-day poor outcomes in patients with aneurysmal subarachnoid haemorrhage: logistic regression analyses.

Factors	Univariable logistic regression analyses^a^				Multivariable logistic regression analyses^b^			
	OR	95% CI for OR		*p* value	AOR	95% CI for AOR		*p* value
		Lower	Upper			Lower	Upper	
Demographics								
Age ≥ 60 years	2.407	1.586	3.654	< 0.001	3.181	1.393	7.262	0.006
Risk factors of aneurysmal SAH								
Hypertension	2.056	1.354	3.122	0.001	NA	NA	NA	NA
Comorbidities								
Diabetes mellitus	3.751	1.669	8.433	0.001	NA	NA	NA	NA
Neuroimaging findings on admission								
Location of blood within the subarachnoid space:								
Basal cistern	2.685	1.728	4.173	< 0.001	NA	NA	NA	NA
Sylvian fissure	2.981	1.125	7.900	0.028	NA	NA	NA	NA
Interhemispheric fissure	1.960	1.207	3.183	0.007	NA	NA	NA	NA
Interpeduncular fossa	3.254	1.995	5.308	< 0.001	2.673	1.106	6.460	0.029
Suprasellar cistern	1.929	1.221	3.047	0.005	NA	NA	NA	NA
Ambient cistern	3.054	1.902	4.906	< 0.001	NA	NA	NA	NA
Quadrigeminal cistern	5.141	3.270	8.085	< 0.001	NA	NA	NA	NA
IVH	3.013	1.847	4.914	< 0.001	NA	NA	NA	NA
ICH	1.860	1.142	3.029	0.013	NA	NA	NA	NA
Aneurysm locations								
PCoA aneurysm	0.554	0.299	1.026	0.061	NA	NA	NA	NA
VA aneurysm	3.341	1.265	8.820	0.015	NA	NA	NA	NA
Severity of aneurysmal SAH on admission								
PAASH grade of III–V	15.823	9.480	26.409	< 0.001	11.023	4.544	26.740	< 0.001
WFNS grade of IV–V	15.770	9.547	26.048	< 0.001	NA	NA	NA	NA
H&H grade of IV–V	14.612	8.887	24.025	< 0.001	NA	NA	NA	NA
Aneurysm repairs and other treatments								
Aneurysm repairs								
No aneurysm repair	Reference			< 0.001	Reference			< 0.001
Endovascular coiling	0.009	0.003	0.027	< 0.001	0.008	0.002	0.032	< 0.001
Surgical clipping	0.020	0.007	0.059	< 0.001	0.015	0.004	0.056	< 0.001
Nimodipine	0.076	0.028	0.202	< 0.001	NA	NA	NA	NA
Complications								
Rebleeding	7.868	2.537	24.396	< 0.001	21.817	4.641	102.548	< 0.001
DCI	7.316	2.848	18.792	< 0.001	17.563	4.618	66.789	< 0.001
Acute hydrocephalus	3.134	2.034	4.830	< 0.001	2.150	0.938	4.931	0.071
Pneumonia	4.091	2.295	7.292	< 0.001	4.182	1.632	10.716	0.003
Constant					0.801			0.753

*AOR* adjusted odds ratio, *CI* confidence interval, *DCI* delayed cerebral ischemia, *H&H* Hunt and Hess, *ICH* intracerebral haemorrhage, *IVH* intraventricular haemorrhage, *mRS* modified Rankin Scale, *NA* not available, *OR* odds ratio, *PAASH* Prognosis on Admission of Aneurysmal Subarachnoid Haemorrhage, *PCoA* posterior communicating artery, *SAH* subarachnoid haemorrhage, *VA* vertebral artery, *WFNS* World Federation of Neurosurgical Societies.

^a^Each variable of the demographics, risk factors for aneurysmal SAH, comorbidities, initial clinical, neuroimaging and laboratory characteristics, the severity of aneurysmal SAH (i.e., the grades of PAASH, WFNS, and H&H grading scales that were either greater than or equal to the cut-off value) on admission, treatments, and complications was analysed in the univariable logistic regression model and was considered in the multivariable logistic regression model if the *p* value was < 0.05 in univariable logistic regression analysis, as well as clinically crucial factors.

^b^All selected variables were included in the multivariable logistic regression model with the stepwise backward elimination method. Variables, then, were deleted stepwise from the full model until all remaining variables were independently associated with poor outcomes.

**Table 6:** Factors associated with 90-day poor outcomes in patients with aneurysmal subarachnoid haemorrhage: logistic regression analyses.

Factors	Univariable logistic regression analyses^a^				Multivariable logistic regression analyses^b^			
	OR	95% CI for OR		*p* value	AOR	95% CI for AOR		*p* value
		Lower	Upper			Lower	Upper	
Demographics								
Age ≥ 60 years	2.581	1.691	3.939	< 0.001	3.495	1.518	8.049	0.003
Risk factors of aneurysmal SAH								
Hypertension	2.133	1.400	3.250	< 0.001	NA	NA	NA	NA
Comorbidities								
Diabetes mellitus	3.986	1.772	8.968	0.001	3.786	0.856	16.746	0.079
Neuroimaging findings on admission								
Location of blood within the subarachnoid space:								
Basal cistern	2.718	1.738	4.251	< 0.001	NA	NA	NA	NA
Sylvian fissure	2.811	1.060	7.454	0.038	NA	NA	NA	NA
Interhemispheric fissure	2.063	1.257	3.385	0.004	NA	NA	NA	NA
Interpeduncular fossa	3.206	1.953	5.264	< 0.001	NA	NA	NA	NA
Suprasellar cistern	1.882	1.186	2.986	0.007	NA	NA	NA	NA
Ambient cistern	2.990	1.852	4.829	< 0.001	NA	NA	NA	NA
Quadrigeminal cistern	4.414	3.431	8.545	< 0.001	2.477	1.056	5.809	0.037
IVH	2.793	1.711	4.559	< 0.001	NA	NA	NA	NA
ICH	1.879	1.151	3.068	0.012	NA	NA	NA	NA
Aneurysm locations								
PCoA aneurysm	0.590	0.318	1.094	0.094	NA	NA	NA	NA
VA aneurysm	3.542	1.341	9.356	0.011	NA	NA	NA	NA
Severity of aneurysmal SAH on admission								
PAASH grade of III–V	14.771	8.894	24.531	< 0.001	10.868	4.281	27.592	< 0.001
WFNS grade of IV–V	15.387	9.291	25.483	< 0.001	NA	NA	NA	NA
H&H grade of IV–V	14.034	8.535	23.076	< 0.001	NA	NA	NA	NA
Aneurysm repairs and other treatments								
Aneurysm repairs								
No aneurysm repair	Reference			< 0.001	Reference			< 0.001
Endovascular coiling	0.011	0.004	0.030	< 0.001	0.016	0.004	0.060	< 0.001
Surgical clipping	0.023	0.009	0.062	< 0.001	0.030	0.008	0.105	< 0.001
Nimodipine	0.071	0.027	0.191	< 0.001	0.193	0.030	1.232	0.082
Complications								
Rebleeding	6.133	2.138	17.594	0.001	27.870	5.748	135.118	< 0.001
DCI	6.245	2.538	15.365	< 0.001	15.430	3.809	62.504	< 0.001
Acute hydrocephalus	3.134	2.028	4.842	< 0.001	2.224	0.976	5.065	0.057
Pneumonia	4.022	2.263	7.148	< 0.001	4.358	1.678	11.321	0.003
Constant					2.253			0.413

*AOR* adjusted odds ratio, *CI* confidence interval, *DCI* delayed cerebral ischemia, *H&H* Hunt and Hess, *ICH* intracerebral haemorrhage, *IVH* intraventricular haemorrhage, *mRS* modified Rankin Scale, *NA* not available, *OR* odds ratio, *PAASH* Prognosis on Admission of Aneurysmal Subarachnoid Haemorrhage, *PCoA* posterior communicating artery, *SAH* subarachnoid haemorrhage, *VA* vertebral artery, *WFNS* World Federation of Neurosurgical Societies.

^a^Each variable of the demographics, risk factors for aneurysmal SAH, comorbidities, initial clinical, neuroimaging and laboratory characteristics, the severity of aneurysmal SAH (i.e., the grades of PAASH, WFNS, and H&H grading scales that were either greater than or equal to the cut-off value) on admission, treatments, and complications was analysed in the univariable logistic regression model and was considered in the multivariable logistic regression model if the *p* value was < 0.05 in univariable logistic regression analysis, as well as clinically crucial factors.

^b^All selected variables were included in the multivariable logistic regression model with the stepwise backward elimination method. Variables, then, were deleted stepwise from the full model until all remaining variables were independently associated with poor outcomes.

## Discussion

The present study revealed that nearly one-third of patients with aneurysmal SAH had poor outcomes on days 30th and 90th after ictus (33.3% and 32.0%, respectively), over one-fifth of whom died within 30 and 90 days of ictus (21.4% and 23.4%, respectively) (Fig. 1 and Table 1). The PAASH, WFNS, and H&H scales all had good discriminatory ability concerning the prognosis of patients on days 30th and 90th after ictus (Figs. 2 and 3, Table S10 as shown in Additional file 1), with no significant differences between the AUROC curves among different test-pairwise (Table 2). Significant differences between the 30- and 90-day mean mRS scores of the adjacent grades were observed more often in the PAASH scale (i.e., grade I vs. II, grade II vs. III) compared to those in the WFNS (i.e., grade IV vs. V) and the H&H scales (i.e., grade IV vs. V) (Table 3). The PAASH scale showed more gradual increases in OR for the poor outcome on days 30th and 90th after ictus, in ascending grades, in the univariable logistic regression analyses, with grade I taken as the reference, compared to the WFNS and H&H scales (Table 4). In contrast to a WFNS grade of IV–V and an H&H grade of IV–V, a PAASH grade of III–V was an independent predictor of the poor outcome on days 30th and 90th after ictus in the multivariable logistic regression analyses (Tables 5 and 6).

The mortality rates of our patients on days 30th and 90th after ictus were lower than the rates reported in previous studies (22–25% and 25–29%, respectively)^5,39^. These differences might be because our cohort is likely to be highly selected as many patients with aneurysmal SAH in Vietnam are not transferred to a central hospital and might die in the local hospital as well as outside of the hospital^40^. Additionally, our study only included patients presenting to the participating hospitals within four days of ictus and excluded patients for whom we could not score admission GCS (e.g., patients intubated and under sedation before arrival at the central hospital). Thus, these factors have resulted in an implicit selection bias and an enrolment and inclusion incompletion of patients in the study database. As a result, our cohort is likely to be underestimated in the poor outcome and mortality rates.

Although there were no significant differences between the AUROC curves among different test-pairwise, the PAASH, WFNS, and H&H scales all had good discriminatory ability concerning the prognosis of patients on days 30th and 90th after ictus. However, significant differences regarding the mean mRS scores on days 30th and 90th after ictus between the adjacent grades were observed more often in the PAASH scale (i.e., grade I vs. II, grade II vs. III) compared to those in the WFNS (i.e., grade IV vs. V) and the H&H scales (i.e., grade IV vs. V). To date, there is no universally accepted scale to assess the clinical condition of these patients at the time of admission^22,41^. Although the WFNS and H&H scales are both widely used in clinical practice and research reports, the WFNS scale is complex to administer because it requires an assessment of both motor function and GCS^21^, and the interobserver agreement for the H&H scale is poor^23,24^. The WFNS scale has two main advantages over the GCS alone. It compresses the GCS into five grades, which may create greater intergrade differences in outcome. It includes the presence of a focal motor deficit axis. However, the amount of additional prognostic power derived from adding this axis is unknown^22^. Our study showed no significant differences regarding the outcomes (mean mRS scores) on days 30th and 90th after ictus between grades I and II, II and III, and IV and V of the WFNS scale. A previous study also showed that the differences in the outcomes between grades II and III failed to reach statistical significance on the WFNS scale^42^. These findings might be due to the lack of formal validation of the WFNS scale, which might lead to occasional overlap between grades (particularly between grades II and III), where the outcomes predicted by the assigned grade may not differ substantially^26,43^. The present study also showed no significant differences regarding the outcomes (mean mRS scores) on days 30th and 90th after ictus between the adjacent grades of the H&H scale except for between grades IV and V. Although the H&H scale is easy to administer, the classifications are arbitrary, some of the terms are vague (e.g., drowsy, stupor, and deep coma), and some patients may present with initial features that defy placement within a single grade^22^. As an example, a rare presentation of SAH may include severe headache (i.e., grade II), normal level of consciousness, and severe hemiparesis (i.e., grade IV). In such cases, the clinician must subjectively decide which of the presenting features is the most important for determining the grade. Therefore, our findings might be accounted for by the poor interobserver agreement and might also contribute to the conflicting data regarding the utility of the H&H scale for prognosis^23,24,26^. The PAASH scale is very easy to apply and is based solely on the GCS, which has a much better interobserver agreement^24^. Unlike the originally-suggested PAASH scale, for which the outcome, defined as the mean Glasgow Outcome Scale (GOS) score, on day 180th after ictus of each grade differs from that of adjacent grades with a high statistical significance^29^, the present study showed that only significant differences in the outcome on days 30th and 90th after ictus between grades I and II, and between grades II and III. This variation might be because of the differences concerning the outcome measures (i.e., GOS vs. mRS) and the time points of outcome evaluation (i.e., on day 180th vs. on days 30th and 90th after ictus) between the two studies. The fact that significant differences regarding the mean mRS scores on days 30th and 90th after ictus between the adjacent grades were observed more often in the PAASH scale compared to those in the WFNS and the H&H scales in our study suggests that the PAASH scale may be preferable to the WFNS and H&H scales.

Previous critiques have identified a caution problem with ORs^44–48^, and a recent literature review has raised this issue again^49^. For example, (1) there is no single OR; instead, any estimated OR is conditional on the data and the model specification; (2) ORs should not be compared across different studies using different samples from different populations; and (3) nor should they be compared across models with different sets of confounding variables^49^. Therefore, we used the univariable logistic regression analyses, with the same grade (i.e., I) taken as the reference, and the multivariable logistic regression analyses, with the same set of confounding variables, to determine the relationships among the grades on the PAASH, WFNS, and H&H scales and the actual outcomes. In our study, the PAASH scale showed a more gradual increase in OR for the poor outcome on days 30th and 90th after ictus, in ascending grades, compared to the WFNS and H&H scales in the univariable logistic regression analyses, with grade I taken as the reference. These findings are consistent with a previously published study^30^, and might be explained by the fact that the same or more number of grades that were significantly associated with the increased risk of poor outcome was observed in the PAASH scale (i.e., grades II, III, IV, and V) compared to the WFNS (i.e., grades II, III, IV, and V) and the H&H scale (i.e., grades III, IV, and V). When we added each SAH grading scale (i.e., PAASH, WFNS, or H&H scale) to the multivariable logistic regression model, with the same set of confounding variables, for predicting the poor outcome on days 30th and 90th after ictus, we also found that a more gradual increase in AOR of the PAASH scale, in ascending grades, compared to those of the WFNS and H&H scales, which might be due to the same or more number of grades that were independently associated with the increased risk of poor outcome was observed in the PAASH scale (i.e., grades III, IV, and V) compared to the WFNS (i.e., grades IV and V) and the H&H scale (i.e., grades III, IV, and V). Therefore, a more gradual increase in effect size (i.e., OR and AOR) of the PAASH scale, in ascending grades, for predicting the poor outcome suggests that it may be preferable to the WFNS and H&H scales.

Although the advances in diagnostic and treatment strategies for aneurysmal SAH have substantially improved the outcomes of hospitalized patients in recent decades^50–53^, predicting the outcome of aneurysmal SAH remains a problematic issue. The clinical condition can vary during the acute phase, and complications occurring during the clinical course and treatments rendered can influence the outcome^31,54,55^. In the present study, complications (e.g., rebleeding, DCI, pneumonia) also accounted for a substantial proportion of patients with aneurysmal SAH and contributed significantly to a high rate of poor outcomes. Nevertheless, grading patients with SAH on admission is crucial for clinical and research purposes. Most grading systems are used in practice to standardize the clinical classification of patients with SAH based only on the initial neurologic examination and the appearance of blood on the initial head CT^20,21,29,56^. Therefore, a scale applied upon admission will never give a 100% perfect prediction for the outcome. In our multivariable logistic regression analyses, in contrast to a WFNS grade of IV–V and an H&H grade of IV–V, a PAASH grade of III–V was an independent predictor of the poor outcome on days 30th and 90th after ictus. Thus, these findings support the superiority of the PAASH scale compared to the WFNS and H&H scales in predicting poor outcomes.

An advantage of the present study was data from many centres, which had little missing data (Table S17 as shown in Additional file 1). However, the present study has some limitations, as follows: *Firstly*, our data are from a selected population of cases that were mainly transferred to the three highest-level public sector hospitals in Vietnam. Therefore, the number of patients with aneurysmal SAH is likely to be considerably higher. *Secondly*, only one clinician or surgeon provided an initial evaluation for each patient in our study; therefore, interobserver variability was not studied. *Finally*, this study only included patients presenting to the participating hospitals within 4 days of ictus and excluded patients for whom admission GCS was unable to be scored (e.g., patients intubated and under sedation before arrival at the central hospital). Therefore, these factors resulted in incomplete enrolment of patients in the database of the study, which may have introduced selection bias. These limitations might account for some differences in figures reported from other countries.

## Conclusions

This study investigated a selected cohort of patients with aneurysmal SAH, a high rate of poor outcomes and a high mortality rate presented to central hospitals in Vietnam. The PAASH, WFNS and H&H scales all have good discriminatory abilities for the prognosis of patients with aneurysmal SAH. Because of the more clearly significant difference between the clinical outcomes of the adjacent grades and the more strong effect size for predicting poor outcomes, the PAASH scale was preferable to the WFNS and H&H scales.

## Supplementary Information

Supplementary Tables.

## Data Availability

All data generated or analysed during this study are included in this published article (and its Supplementary Information files).
