# Supplementary material for: Predictive validity of the prognosis on admission aneurysmal subarachnoid haemorrhage scale for the outcome of patients with aneurysmal subarachnoid haemorrhage
Source: Sci Rep. 2023 Apr 25;13:6721. doi: 10.1038/s41598-023-33798-5 (PMC10130082; doi:10.1038/s41598-023-33798-5)
Supplement: Supplementary file 1 — Supplementary Tables. [file 41598_2023_33798_MOESM1_ESM.pdf]

SUPPLEMENTARY RESULTS

Article title: Validation of the accuracy of the Prognosis on Admission Aneurysmal Subarachnoid Haemorrhage scale for predicting the outcomes of patients with aneurysmal subarachnoid haemorrhage

TABLE OF CONTENT

Table S1. Criteria per grading scale for patients with subarachnoid haemorrhage

Table S2. Demographic and baseline characteristics of patients with aneurysmal subarachnoid haemorrhage according to neurologic function on day 30th after ictus

Table S3. Clinical presentation, neuroimaging findings, and laboratory investigations of patients with aneurysmal subarachnoid haemorrhage according to neurologic function on day 30th after ictus

Table S4. Initial severity of aneurysmal subarachnoid haemorrhage in patients with aneurysmal subarachnoid haemorrhage according to neurologic function on day 30th after ictus

Table S5. Management, complications, and outcomes of patients with aneurysmal subarachnoid haemorrhage according to neurologic function on day 30th after ictus

Table S6. Demographic and baseline characteristics of patients with aneurysmal subarachnoid haemorrhage according to neurologic function on day 90th after ictus

Table S7. Clinical presentation, neuroimaging findings, and laboratory investigations of patients with aneurysmal subarachnoid haemorrhage according to neurologic function on day 90th after ictus

Table S8. Initial severity of aneurysmal subarachnoid haemorrhage in patients with aneurysmal subarachnoid haemorrhage according to neurologic function on day 90th after ictus

Table S9. Management, complications, and outcomes of patients with aneurysmal subarachnoid haemorrhage according to neurologic function on day 90th after ictus

Table S10. Summary table of various diagnostic indices and AUROC of the PAASH, WFNS, and H&H scales for predicting the poor outcome (mRS of 4 to 6) after ictus in patients with aneurysmal SAH

Table S11. Factors associated with poor outcome (mRS of 4 to 6) on day 30th after ictus in patients with aneurysmal subarachnoid haemorrhage (the exposure variable was defined as the originally-suggested 5-category PAASH grading scale)

Table S12. Factors associated with poor outcome (mRS of 4 to 6) on day 30th days ictus in patients with aneurysmal subarachnoid haemorrhage (the exposure variable was defined as the originally-suggested 5-category WFNS grading scale)

Table S13. Factors associated with poor outcome (mRS of 4 to 6) on day 30th after ictus in patients with aneurysmal subarachnoid haemorrhage (the exposure variable was defined as the originally-suggested 5-category H&H grading scale)

Table 14. Factors associated with poor outcome (mRS of 4 to 6) on day 90th after ictus in patients with aneurysmal subarachnoid haemorrhage (the exposure variable was defined as the originally-suggested 5-category PAASH grading scale)

Table S15. Factors associated with poor outcome (mRS of 4 to 6) on day 90th after ictus in patients with aneurysmal subarachnoid haemorrhage (the exposure variable was defined as the originally-suggested 5-category WFNS grading scale)

Table S16. Factors associated with poor outcome (mRS of 4 to 6) on day 90th after ictus in patients with aneurysmal subarachnoid haemorrhage (the exposure variable was defined as the originally-suggested 5-category H&H grading scale)

Table S17. Breakdown of missing data

Table S1. Criteria per grading scale for patients with subarachnoid haemorrhage

Numerical SAH grading scales (point)	Descriptive SAH grading scales			
	Grade	H&H grading scale (Hunt WE. *J Neurosurg*. 1968)	WFNS grading scale (WFNS. *J Neurosurg*. 1988)	PAASH grading scale (Takagi K. *J Neurosurg*. 1999)
1	I	Asymptomatic or mild headache and slight nuchal rigidity	GCS score of 15, no motor deficit	GCS score of 15
2	II	Severe headache, stiff neck, no neurologic deficit except cranial nerve palsy	GCS score of 13 to 14, no motor deficit	GCS score of 11 to 14
3	III	Drowsy or confused, mild focal neurologic deficit	GCS score of 13 to 14, motor deficit	GCS score of 8 to 10
4	IV	Stuporous, moderate or severe hemiparesis	GCS score of 7 to 12	GCS score of 4 to 7
5	V	Coma, decerebrate posturing	GCS score of 3 to 6	GCS score of 3

Abbreviations

GCS: Glasgow coma scale; **H&H**: Hunt and Hess; **PAASH**: Prognosis on Admission of Aneurysmal Subarachnoid Haemorrhage; **SAH**, subarachnoid haemorrhage; **WFNS**: World Federation of Neurological Surgeons

Table S2. Demographic and baseline characteristics of patients with aneurysmal subarachnoid haemorrhage according to neurologic function on day 30th after ictus

	All cases	mRS of 0 to 3	mRS of 4 to 6	p-value[a]
Prehospital setting	n=415	n=277	n=138	
Transferred from local hospitals, n (%)	345 (83.1)	234 (84.5)	111 (80.4)	0.300
Hospital taken to, n (%)	n=415	n=277	n=138	0.105
Vietnam-Germany Friendship	30 (7.2)	25 (9.0)	5 (3.6)	
Bach Mai	358 (86.3)	236 (85.2)	122 (88.4)	
Hanoi Medical University	27 (6.5)	16 (5.8)	11 (8.0)	
**Demographics**	n=415	n=277	n=138	
Age (year), median (IQR)	57.0 (48.0-67.0)	56.0 (46.5-64.0)	63.5 (52.0-72.0)	<0.001***

Gender (male), no. (%)	198 (47.7)	131 (47.3)	67 (48.6)	0.809
**Risk factors of aneurysmal SAH**	n=415	n=277	n=138	
Cigarette smoking, no. (%)	159 (38.3)	102 (36.8)	57 (41.3)	0.376
Hypertension, no. (%), n=413	163 (39.5)	93 (33.7)	70 (51.1)	0.001
Genetic risk, no. (%)	6 (1.4)	5 (1.8)	1 (0.7)	0.668*
Alcohol consumption, no. (%), n=401	182 (45.4)	117 (44.2)	65 (47.8)	0.448
Sympathomimetic drugs, no. (%), n=413	3 (0.7)	3 (1.1)	0 (0.0)	0.554*
Oestrogen deficiency, no. (%), n=213[b]	33 (15.5)	20 (14.1)	13 (18.3)	0.422
Antithrombotic therapy, no. (%)	4 (1.0)	1 (0.4)	3 (2.2)	0.109*
Elevated total cholesterol, no. (%)	11 (2.7)	6 (2.2)	5 (3.6)	0.517*
**Comorbidities**	n=415	n=277	n=138	
Cerebrovascular disease, no. (%)	18 (4.3)	10 (3.6)	8 (5.8)	0.303
Chronic cardiac failure, no. (%)	7 (1.7)	3 (1.1)	4 (2.9)	0.228*
Coronary artery disease/MI, no. (%)	7 (1.7)	3 (1.1)	4 (2.9)	0.228*
COPD/Asthma, no. (%)	5 (1.2)	3 (1.1)	2 (1.4)	>0.999*
Active neoplasm, no. (%)	7 (1.7)	5 (1.8)	2 (1.4)	>0.999*
Chronic renal failure, no. (%)	3 (0.7)	2 (0.7)	1 (0.7)	>0.999*
Ulcer disease, no. (%)	4 (1.0)	2 (0.7)	2 (1.4)	0.603*
Diabetes mellitus, no. (%)	27 (6.5)	10 (3.6)	17 (12.3)	0.001
Haematological disease, no. (%)	2 (0.5)	1 (0.4)	1 (0.7)	>0.999*

[a] Comparison between mRS of 0 to 3 and mRS of 4 to 6 using Chi-squared test; *Fisher's exact test; **Mann–Whitney U test; *** Independent Samples T test

[b] Exclude 198 male patients and 4 female patients with missing data

Abbreviations: **COPD**: chronic obstructive pulmonary disease; **IQR**: interquartile range; **MI**: myocardial ischemia; **mRS**: modified Rankin Scale; no.: number; **SAH**: subarachnoid haemorrhage.

Table S3. Clinical presentation, neuroimaging findings, and laboratory investigations of patients with aneurysmal subarachnoid haemorrhage according to neurologic function on day 30th after ictus

	All cases	mRS of 0 to 3	mRS of 4 to 6	p-value[a]
**Onset symptoms**	n=415	n=277	n=138	
Sudden-onset, severe headache, no. (%)	337 (81.2)	252 (91.0)	85 (61.6)	<0.001
Vomiting, no. (%)	248 (59.8)	189 (68.2)	59 (42.8)	<0.001
Neck pain or stiffness, no. (%)	111 (26.7)	83 (30.0)	28 (20.3)	0.036
Photophobia, no. (%)	15 (3.6)	13 (4.7)	2 (1.4)	0.160*
Blurred or double vision, no. (%)	12 (2.9)	10 (3.6)	2 (1.4)	0.352*
Brief loss of consciousness, no. (%)	146 (35.2)	62 (22.4)	84 (60.9)	<0.001
Seizures, no. (%)	18 (4.3)	10 (3.6)	8 (5.8)	<0.303
**Clinical presentation on admission**	n=415	n=277	n=138	
GCS score, median (IQR)	14.0 (10.0-15.0)	15.0 (14.0-15.0)	8.0 (6.0-12.25)	<0.001**
Focal deficits, no. (%)	315 (75.9)	197 (71.1)	118 (85.5)	0.001
Focal signs, n (%)	n=315	n=197	n=118	
Third nerve palsy	8 (2.5)	4 (2.0)	4 (3.4)	0.479*
Sixth nerve palsy	1 (0.3)	1 (0.5)	0 (0.0)	>0.999*
Hemiparesis	59(18.7)	33 (16.8)	26 (22.0)	0.245
Aphasia	10(3.2)	5 (2.5)	5 (4.2)	0.510*
Bilateral leg weakness	2(0.6)	1 (0.5)	1 (0.8)	>0.999*
Ophthalmoplegia	1(0.3)	1 (0.5)	0 (0.0)	>0.999*
Impaired level of consciousness, n=314	154 (49.0)	58 (29.4)	96 (82.1)	<0.001
Brainstem signs	5 (1.6)	1 (0.5)	4 (3.4)	0.067*
Neck stiffness	254 (80.6)	168 (85.3)	86 (72.9)	0.007
**Neuroimaging findings on admission**	n=415	n=277	n=138	
Blood filling the subarachnoid space, no. (%)				
Basal cistern, n=411	228 (55.5)	132 (47.8)	96 (71.1)	<0.001
Sylvian fissure, n=413	380 (92.0)	248 (89.9)	132 (96.4)	0.022
Interhemispheric fissure, n=412	291 (70.6)	183 (66.3)	108 (79.4)	0.006
Interpeduncular fossa, n=412	266 (64.6)	156 (56.5)	110 (80.9)	<0.001
Suprasellar cistern,	270 (65.5)	168 (60.9)	102 (75.0)	0.005

n=412				
Ambient cistern, n=412	258 (62.6)	151 (54.7)	107 (78.7)	<0.001
Quadrigeminal cistern, n=412	126 (30.6)	52 (18.8)	74 (54.4)	<0.001
IVH, n (%)	275 (66.3)	163 (58.8)	112 (81.2)	<0.001
ICH, n (%)	85 (20.5)	47 (17.0)	38 (27.5)	0.012
ICH volume (mL), mean (SD), n=85	22.6 (22.82)	17.55 (16.98)	28.86 (27.40)	0.097**
Subdural haemorrhage, n (%)	21 (5.1)	9 (3.2)	12 (8.7)	0.017
Hydrocephalus, n (%)	133(32.0)	66 (23.8)	67 (48.6)	<0.001
Evans' index, mean (SD), n=392	0.27 (0.07)	0.27 (0.07)	0.29 (0.08)	0.002**
Hypodense lesions on computed tomography, n (%), n=413	26 (6.3)	9 (3.3)	17 (12.4)	<0.001
Aneurysm site, n (%)				
Internal carotid artery (ICA)	84 (20.2)	55 (19.9)	29 (21.0)	0.782
Ophtalmic segment of the ICA (OphIC)	3 (0.7)	3 (1.1)	0 (0.0)	0.554*
Cavernous segment of the ICA (cIC)	7 (1.7)	5 (1.8)	2 (1.4)	>0.999*
Anterior choroidal artery segment of the ICA (AchIC)	1 (0.2)	1 (0.4)	0 (0.0)	>0.999*
Posterior communicating artery (PCoA)	65 (15.7)	50 (18.1)	15 (10.9)	0.058
Anterior cerebral artery (ACA)	31 (7.5)	21 (7.6)	10 (7.2)	0.903
Anterior communicating artery (AcoA)	130 (31.3)	85 (30.7)	45 (32.6)	0.691
Middle cerebral artery (MCA)	85 (20.5)	62 (22.4)	23 (16.7)	0.174
Posterior cerebral artery (PCA)	1 (0.2)	1 (0.4)	0 (0.0)	>0.999*
Vertebral artery (VA)	18 (4.3)	7 (2.5)	11 (8.0)	0.010
Superior cerebellar artery (SCA)	0 (0.0)	0 (0.0)	0 (0.0)	NA
Posterior inferior cerebellar artery (PICA)	9 (2.2)	5 (1.8)	4 (2.9)	0.488*
Anterior inferior cerebellar artery	0 (0.0)	0 (0.0)	0 (0.0)	NA

(AICA)				
Basilar artery (BA)	15 (3.6)	9 (3.2)	6 (4.3)	0.584*
**Admission laboratory investigations**				
Platelets (G/L), mean (SD), n=408	261.47 (76.04)	257.86 (72.65)	268.7 (82.20)	0.217**
PT-INR, mean (SD), n=398	1.03 (0.51)	1.03 (0.62)	1.04 (0.17)	0.036**

[a] Comparison between mRS of 0 to 3 and mRS of 4 to 6 using Chi-squared test; *Fisher's exact test; **Mann–Whitney U test.

Abbreviations: **GCS**: Glasgow coma scale; **ICH**: intracerebral haemorrhage; **IQR**: interquartile range; **IVH**: intraventricular haemorrhage; **mRS**: modified Rankin Scale; **no.**: number; **PT-INR**: prothrombin time with international normalized ratio; **SD**: standard deviation.

Table S4. Initial severity of aneurysmal subarachnoid haemorrhage in patients with aneurysmal subarachnoid haemorrhage according to neurologic function on day 30th after ictus

	All cases (n=415)	mRS of 0 to 3 (n=277)	mRS of 4 to 6 (n=138)	p-value[a]
PAASH score[b], median (IQR)	2.0 (1.0-3.0)	1.0 (1.0-2.0)	3.0 (2.0-4.0)	<0.001**
PAASH scale, no. (%)				<0.001
Grade I	204 (49.2)	185 (66.8)	19 (13.8)	
Grade II	86 (20.7)	60 (21.7)	26 (18.8)	
Grade III	53 (12.8)	21 (7.6)	32 (23.2)	
Grade IV	63 (15.2)	10 (3.6)	53 (38.4)	
Grade V	9 (2.2)	1 (0.4)	8 (5.8)	
WFNS score[b], median (IQR)	2.0 (1.0-4.0)	1.0 (1.0-2.0)	4.0 (3.75-5.0)	<0.001**
WFNS scale, no. (%)				<0.001
Grade I	204 (49.2)	185 (66.8)	19 (13.8)	
Grade II	48 (11.6)	37 (13.4)	11 (8.0)	
Grade III	14 (3.4)	10 (3.6)	4 (2.9)	
Grade IV	99 (23.9)	38 (13.7)	61 (44.2)	
Grade V	50 (12.0)	7 (2.5)	43 (31.2)	
H&H score[b], median (IQR)	2.0 (2.0-4.0)	2.0 (2.0-3.0)	5.0 (3.0-5.0)	<0.001**
H&H scale, no. (%)				<0.001
Grade I	45 (10.8)	40 (14.4)	5 (3.6)	
Grade II	168 (40.5)	153 (55.2)	15 (10.9)	
Grade III	62 (14.9)	43 (15.5)	19 (13.8)	
Grade IV	48 (11.6)	25 (9.0)	23 (16.7)	

Grade V	92 (22.2)	16 (5.8)	76 (55.1)	
Fisher score[b], median (IQR), n=414	4.0 (3.0-4.0)	4.0 (3.0-4.0)	4.0 (4.0-4.0)	<0.001[**]
Fisher scale, no. (%)	n=414	n=276	n=138	<0.001[*]
Group 1	2 (0.5)	1 (0.4)	1 (0.7)	
Group 2	24 (5.8)	23 (8.3)	1 (0.7)	
Group 3	98 (23.7)	80 (29.0)	18 (13.0)	
Group 4	290 (70.0)	172 (62.3)	118 (85.5)	

[a] Comparison between mRS of 0 to 3 and mRS of 4 to 6 using Chi-squared test; *Fisher's exact test; **Mann–Whitney U test.

[b] The descriptive SAH grading scales (i.e., the PAASH, WFNS, and H&H scales) were converted to the numerical SAH grading scales in ascending order (see Table S1, as shown in Additional file 1, for additional information).

Abbreviations: **H&H**: Hunt and Hess; **IQR**: interquartile range; **mRS**: modified Rankin Scale; **no.**: number; **PAASH**: Prognosis on Admission of Aneurysmal Subarachnoid Haemorrhage; **SD**: standard deviation; **WFNS**: World Federation of Neurological Surgeons.

Table S5. Management, complications, and outcomes of patients with aneurysmal subarachnoid haemorrhage according to neurologic function on day 30th after ictus

	All cases (n=415)	mRS of 0 to 3 (n=277)	mRS of 4 to 6 (n=138)	p-value[a]
**Aneurysm repairs and other treatments**	n=415	n=277	n=138	
No aneurysm repair, no. (%)	74 (17.8)	4 (1.4)	70 (50.7)	<0.001
Endovascular coiling, no. (%)	169 (40.7)	146 (52.7)	23 (16.7)	<0.001
Surgical clipping, no. (%)	172 (41.5)	127 (45.8)	45 (32.6)	0.010
Surgical hematoma evacuation[b], no. (%)	44 (10.6)	21 (7.6)	23 (16.7)	0.005
EVD, no. (%), n=414	43 (10.4)	17 (6.2)	26 (18.8)	<0.001
IVF, no. (%)	3 (0.7)	1 (0.4)	2 (1.4)	0.258[*]
Nimodipine, no. (%), n=363	331 (91.2)	235 (97.9)	96 (78.0)	<0.001
**Complications**				
Rebleeding, no. (%), n=411	18 (4.4)	4 (1.4)	14 (10.4)	<0.001
Early rebleeding, no. (%), n=13				
Late rebledding, no. (%), n=13				
DCI, no. (%), n=409	25 (6.1)	6 (2.2)	19 (14.1)	<0.001
Acute hydrocephalus, no.	136 (32.8)	67 (24.2)	69 (50.0)	<0.001

(%)				
Hyponatremia, no. (%)	71 (17.1)	45 (16.2)	26 (18.8)	0.508
Seizures, no. (%)	53 (12.8)	36 (13.0)	17 (12.3)	0.846
Chronic hydrocephalus, no. (%), n=311	8 (2.6)	6 (2.6)	2 (2.4)	>0.999*
Ventriculitis, no. (%), n=369	13 (3.5)	5 (2.0)	8 (6.5)	0.037*
Pneumonia, no. (%)	58 (14.0)	22 (7.9)	36 (26.1)	<0.001
Urinary tract infection, no. (%)	9 (2.2)	4 (1.4)	5 (3.6)	0.166*
**Clinical time course**	n=415	n=277	n=138	
Ictus to hospital arrival (hour), no. (%), n=408				0.060
≤ 24 hours	212 (52.0)	131 (48.2)	81 (59.6)	
>24–72 hours	188 (46.0)	134 (49.2)	54 (39.7)	
>72 hours	8 (2.0)	7 (2.6)	1 (0.7)	
Length of hospitalization (days), mean (SD)	10.14 (9.85)	11.11 (9.41)	8.2 (10.46)	<0.001**
**Clinical outcomes**	n=415	n=277	n=138	
Hospital discharge, no. (%)	119 (28.7)	115 (41.5)	4 (2.9)	<0.001
Transferred to another hospital, no. (%)	252 (60.7)	162 (58.5)	90 (65.2)	0.186
Discharged to die, no. (%)	33 (8.0)	1 (0.4)	32 (23.2)	<0.001
Deaths:				
Died in hospital, no. (%)	71 (17.1)	0 (0.0)	71 (51.4)	<0.001
Died within 30 days of ictus, no. (%)	89 (21.4)	0 (0.0)	89 (64.5)	<0.001
Neurological function:				
mRS score at hospital discharge, median (IQR)	1.0 (1.0-5.0)	1.0 (1.0-1.0)	5.0 (5.0-5.0)	<0.001**
mRS at hospital discharge, no. (%)				<0.001
Good (mRS of 0 to 3)	266 (64.1)	262 (94.6)	4 (2.9)	
Poor (mRS of 4 to 6)	149 (35.9)	15 (5.4)	134 (97.1)	

[a] Comparison between mRS of 0 to 3 and mRS of 4 to 6 using Chi-squared test; *Fisher's exact test; **Mann–Whitney U test.

[b] Surgical haematoma evacuation was defined as any surgical procedure evacuating epidural, subdural, intraventricular, or intraparenchymal haematoma, such as decompressive craniotomy, open craniotomy, or minimally invasive surgery.

Abbreviations: **DCI**: delayed cerebral ischemia; **EVD**: external ventricular drainage; **IQR**: interquartile range; **IVF**: intraventricular fibrinolysis; **mRS**: modified Rankin Scale; **no.**: number; **SD**: standard deviation.

Table S6. Demographic and baseline characteristics of patients with aneurysmal subarachnoid haemorrhage according to neurologic function on day 90th after ictus

	All cases	mRS of 0 to 3	mRS of 4 to 6	p-value[a]
Prehospital setting	n=415	n=282	n=133	
Transferred from local hospitals, no. (%)	345 (83.1)	238 (84.4)	107 (80.5)	0.316
Hospital taken to, n (%)	n=415	n=282	n=133	0.052
Vietnam-Germany Friendship	30 (7.2)	26 (9.2)	4 (3.0)	
Bach Mai	358 (86.3)	240 (85.1)	118 (87.1)	
Hanoi Medical University	27 (6.5)	16 (5.7)	11 (8.3)	
**Demographics**	n=415	n=282	n=133	
Age (year), median (IQR)	57.0 (48.0-67.0)	56.0 (46.0-64.0)	64.0 (53.0-72.5)	<0.001***
Gender (male), no. (%)	198 (47.7)	134 (47.5)	64 (48.1)	0.909
**Risk factors of aneurysmal SAH**	n=415	n=282	n=133	
Cigarette smoking, no. (%)	159 (38.3)	103 (36.5)	56 (42.1)	0.275
Hypertension, no. (%), n=413	163 (39.5)	94 (33.6)	69 (51.9)	<0.001
Genetic risk, no. (%)	6 (1.4)	5 (1.8)	1 (0.8)	0.669*
Alcohol consumption, no. (%), n=401	182 (45.4)	119 (44.1)	63 (48.1)	0.449
Sympathomimetic drugs, no. (%), n=413	3 (0.7)	3 (1.1)	0	0.554*
Oestrogen deficiency, no. (%), n=213[b]	33 (15.5)	20 (14.1)	13 (18.3)	0.422
Antithrombotic therapy, no. (%)	4 (1.0)	1 (0.4)	3 (2.3)	0.099*
Elevated total cholesterol, no. (%)	11 (2.7)	6 (2.1)	5 (3.8)	0.340*
**Comorbidities**	n=415	n=282	n=133	
Cerebrovascular disease, no. (%)	18 (4.3)	10 (3.5)	8 (6.0)	0.249
Chronic cardiac failure, no. (%)	7 (1.7)	3 (1.1)	4 (3.0)	0.218*
Coronary artery disease/MI, no. (%)	7 (1.7)	3 (1.1)	4 (3.0)	0.218*
COPD/Asthma, no. (%)	5 (1.2)	3 (1.1)	2 (1.5)	0.657*
Active neoplasm, no. (%)	7 (1.7)	5 (1.8)	2 (1.5)	>0.999*
Chronic renal failure, no.	3 (0.7)	2 (0.7)	1 (0.8)	>0.999*

(%)				
Ulcer disease, no. (%)	4 (1.0)	2 (0.7)	2 (1.5)	0.596[*]
Diabetes mellitus, no. (%)	27 (6.5)	10 (3.5)	17 (12.8)	<0.001
Haematological disease, no. (%)	2 (0.5)	1 (0.4)	1(0.8)	0.539[*]

[a]Comparison between mRS of 0 to 3 and mRS of 4 to 6 using Chi-squared test; *Fisher's exact test; **Mann–Whitney U test; *** Independent Samples T test

[b]Exclude 198 male patients and 4 female patients with missing data

Abbreviations: **COPD**: chronic obstructive pulmonary disease; **IQR**: interquartile range; **MI**: myocardial ischemia; **mRS**: modified Rankin Scale; no.: number; **SAH**: subarachnoid haemorrhage.

Table S7. Clinical presentation, neuroimaging findings, and laboratory investigations of patients with aneurysmal subarachnoid haemorrhage according to neurologic function on day 90th after ictus

	All cases	mRS of 0 to 3	mRS of 4 to 6	p-value[a]
**Onset symptoms**	n=415	n=282	n=133	
Sudden-onset, severe headache, no. (%)	337 (81.2)	255 (90.4)	82 (61.7)	<0.001
Vomiting, no. (%)	248 (59.8)	190 (67.4)	58 (43.6)	<0.001
Neck pain or stiffness, no. (%)	111 (26.7)	84 (29.8)	27 (20.3)	0.042
Photophobia, no. (%)	15 (3.6)	13 (4.6)	2 (1.5)	0.160
Blurred or double vision, no. (%)	12 (2.9)	10 (3.5)	2 (1.5)	0.353
Brief loss of consciousness, no. (%)	146 (35.2)	65 (23.0)	81 (60.9)	<0.001
Seizures, no. (%)	18 (4.3)	10 (3.5)	8 (6.0)	0.249
**Clinical presentation on admission**	n=415	n=282	n=133	
GCS score, median (IQR)	14 (10.0-15.0)	15 (14.0-15.0)	8 (6.0-12.0)	<0.001[**]
Focal deficits, no. (%)	315 (75.9)	202 (71.6)	113 (85.0)	0.003
Focal signs, n (%)	n=315	n=202	n=113	
Third nerve palsy	8 (2.5)	5 (2.5)	3 (2.7)	>0.999[*]
Sixth nerve palsy	1 (0.3)	1 (0.5)	0 (0.0)	>0.999[*]
Hemiparesis	59 (18.7)	33 (16.3)	26 (23.0)	0.145
Aphasia	10 (3.2)	5 (2.5)	5 (4.4)	0.339[*]
Bilateral leg weakness	2 (0.6)	1 (0.5)	1 (0.9)	>0.999[*]
Ophthalmoplegia	1 (0.3)	1 (0.5)	0 (0.0)	>0.999[*]
Impaired level of consciousness, n=314	154 (49.0)	62 (30.7)	92 (82.1)	<0.001
Brainstem signs	5 (1.6)	1 (0.5)	4 (3.5)	0.058[*]
Neck stiffness	254 (80.6)	171 (84.7)	83 (73.5)	0.016
**Neuroimaging findings**	n=415	n=282	n=133	

**on admission**				
Location of blood within the subarachnoid space, no. (%)				
Basal cistern, n=411	228 (55.5)	135 (48.0)	93 (71.5)	<0.001
Sylvian fissure, n=413	380 (92.0)	253 (90.0)	127 (96.2)	0.031
Interhemispheric fissure, n=412	291 (70.6)	186 (66.2)	105 (80.2)	0.004
Interpeduncular fossa, n=412	266 (64.6)	160 (56.9)	106 (80.9)	<0.001
Suprasellar cistern, n=412	270 (65.5)	172 (61.2)	98 (74.8)	0.007
Ambient cistern, n=412	258 (62.6)	155 (55.2)	103 (78.6)	<0.001
Quadrigeminal cistern, n=412	126 (30.6)	53 (18.9)	73 (55.7)	<0.001
IVH, no. (%)	275 (66.3)	168 (59.6)	107 (80.5)	<0.001
ICH, no. (%)	85 (20.5)	48 (17.0)	37 (27.8)	0.011
ICH volume (mL), mean (SD), n=85	22.6 (22.82)	17.79 (16.88)	28.85 (27.78)	0.134**
Subdural haemorrhage, no. (%)	21(5.1)	9 (3.2)	12 (9.0)	0.011
Hydrocephalus, no. (%)	133(32.0)	67 (23.8)	66 (49.6)	<0.001
Evans' index, mean (SD), n=392	0.27 (0.07)	0.26 (0.07)	0.29 (0.08)	0.001**
Hypodense lesions on computed tomography, no. (%), n=413	26 (6.3)	9 (3.2)	17 (12.9)	<0.001
Aneurysm site, n (%)				
Internal carotid artery (ICA)	84 (20.2)	56 (19.9)	28 (21.1)	0.777
Ophtalmic segment of the ICA (OphIC)	3 (0.7)	3 (1.1)	0 (0.0)	0.554*
Cavernous segment of the ICA (cIC)	7 (1.7)	5 (1.8)	2 (1.5)	>0.999*
Anterior choroidal artery segment of the ICA (AchIC)	1 (0.2)	1 (0.4)	0 (0.0)	>0.999*
Posterior communicating artery (PCoA)	65 (15.7)	50 (17.7)	15 (11.3)	0.091
Anterior cerebral artery (ACA)	31 (7.5)	21 (7.4)	10 (7.5)	0.979
Anterior communicating artery	130 (31.3)	86 (30.5)	44 (33.1)	0.596

(AcoA)				
Middle cerebral artery (MCA)	85 (20.5)	64 (22.7)	21 (15.8)	0.104
Posterior cerebral artery (PCA)	1 (0.2)	1 (0.4)	0 (0.0)	>0.999*
Vertebral artery (VA)	18 (4.3)	7 (2.5)	11 (8.3)	0.007
Superior cerebellar artery (SCA)	0 (0.0)	0 (0.0)	0 (0.0)	NA
Posterior inferior cerebellar artery (PICA)	9 (2.2)	5 (1.8)	4 (3.0)	0.476*
Anterior inferior cerebellar artery (AICA)	0 (0.0)	0 (0.0)	0 (0.0)	NA
Basilar artery (BA)	15 (3.6)	10 (3.5)	5 (3.8)	>0.999*
**Admission laboratory investigations**				
Platelets (G/L), mean (SD), n=408	261.47 (76.04)	257.41 (72.27)	270.07 (83.10	0.132**
PT-INR, mean (SD), n=398	1.03 (0.51)	1.03 (0.61)	1.03 (0.17)	0.042**

[a]Comparison between mRS of 0 to 3 and mRS of 4 to 6 using Chi-squared test; *Fisher's exact test; **Mann–Whitney U test.

Abbreviations: **GCS**: Glasgow coma scale; **ICH**: intracerebral haemorrhage; **IQR**: interquartile range; **IVH**: intraventricular haemorrhage; **mRS**: modified Rankin Scale; **no.**: number; **PT-INR**: prothrombin time with international normalized ratio; **SD**: standard deviation.

Table S8. Initial severity of aneurysmal subarachnoid haemorrhage in patients with aneurysmal subarachnoid haemorrhage according to neurologic function on day 90th after ictus

	All cases (n=415)	mRS of 0 to 3 (n=282)	mRS of 4 to 6 (n=133)	p-value[a]
PAASH score[b], median (IQR)	2.0 (1.0-3.0)	1.0 (1.0-2.0)	3.0 (2.0-4.0)	<0.001**
PAASH scale, no. (%)				<0.001
Grade I	204 (49.2)	186 (66.0)	18 (13.5)	
Grade II	86 (20.7)	61 (21.6)	25 (18.8)	
Grade III	53 (12.8)	23 (8.2)	30 (22.6)	
Grade IV	63 (15.2)	11 (3.9)	52 (39.1)	
Grade V	9 (2.2)	1 (0.4)	8 (6.0)	
WFNS score[b], median (IQR)	2.0 (1.0-4.0)	1.0 (1.0-2.0)	4.0 (4.0-5.0)	<0.001**
WFNS scale, no. (%)				<0.001
Grade I	204 (49.2)	186 (66.0)	18 (13.5)	

Grade II	48 (11.6)	38 (13.5)	10 (7.5)	
Grade III	14 (3.4)	10 (3.5)	4 (3.0)	
Grade IV	99 (23.9)	41 (14.5)	58 (43.6)	
Grade V	50 (12.0)	7 (2.5)	43 (32.3)	
H&H score[b], median (IQR)	2.0 (2.0-4.0)	2.0 (2.0-3.0)	5.0 (3.0-5.0)	<0.001**
H&H scale, no. (%)				<0.001
Grade I	45 (10.8)	40 (12.2)	5 (3.8)	
Grade II	168 (40.5)	154 (54.6)	14 (10.5)	
Grade III	62 (14.9)	44 (15.6)	18 (13.5)	
Grade IV	48 (11.6)	26 (9.2)	22 (16.5)	
Grade V	92 (22.2)	18 (6.4)	74 (55.6)	
Fisher score[b], median (IQR), n=414	4.0 (3.0-4.0)	4.0 (3.0-4.0)	4.0 (4.0-4.0)	<0.001**
Fisher scale, no. (%)	n=414	n=281	n=133	<0.001*
Group 1	2 (0.5)	1 (0.4)	1 (0.8)	
Group 2	24 (5.8)	23 (8.2)	1 (0.8)	
Group 3	98 (23.7)	80 (28.5)	18 (13.5)	
Group 4	290 (70.0)	177 (63.0)	113 (85.0)	

[a] Comparison between mRS of 0 to 3 and mRS of 4 to 6 using Chi-squared test; *Fisher's exact test; **Mann–Whitney U test.

[b] The descriptive SAH grading scales (i.e., the PAASH, WFNS, and H&H scales) were converted to the numerical SAH grading scales in ascending order (see Table S1, as shown in Additional file 1, for additional information).

Abbreviations: **H&H**: Hunt and Hess; **IQR**: interquartile range; **mRS**: modified Rankin Scale; **no.**: number; **PAASH**: Prognosis on Admission of Aneurysmal Subarachnoid Haemorrhage; **SD**: standard deviation; **WFNS**: World Federation of Neurological Surgeons.

Table S9. Management, complications, and outcomes of patients with aneurysmal subarachnoid haemorrhage according to neurologic function on day 90th after ictus

	All cases	mRS of 0 to 3	mRS of 4 to 6	p-value[a]
**Aneurysm repairs and other treatments**	n=415	n=282	n=133	
No aneurysm repair, no. (%)	74 (17.8)	5 (1.8)	69 (51.9)	<0.001
Endovascular coiling, no. (%)	169 (40.7)	147 (52.1)	22 (16.5)	<0.001
Surgical clipping, no. (%)	172 (41.5)	130 (46.1)	42 (31.6)	0.005
Surgical hematoma evacuation[b], no. (%)	44 (10.6)	23 (8.2)	21 (15.8)	0.018
EVD, no. (%), n=414	43 (10.4)	18 (6.4)	25 (18.8)	<0.001
IVF, no. (%)	3 (0.7)	1 (0.4)	2 (1.5)	0.242*

Nimodipine, no. (%), n=363	331 (91.2)	239 (98.0)	92 (77.3)	<0.001
**Complications**	n=415	n=282	n=133	
Rebleeding, no. (%), n=411	18 (4.4)	5 (1.8)	13 (10.0)	<0.001
Early rebleeding, no. (%), n=13	1 (7.7)	0 (0.0)	1 (8.3)	>0.999*
Late rebledding, no. (%), n=13	12 (92.3)	1 (100)	11 (91.7)	>0.999*
DCI, no. (%), n=409	25 (6.1)	7 (2.5)	18 (13.8)	<0.001
Acute hydrocephalus, no. (%)	136 (32.8)	69 (24.5)	67 (50.4)	<0.001
Hyponatremia, no. (%)	71 (17.1)	46 (16.3)	25 (18.8)	0.577
Seizures, no. (%)	53 (12.8)	37 (13.1)	16 (12.0)	0.756
Chronic hydrocephalus, no. (%), n=311	8 (2.6)	6 (2.6)	2 (2.5)	>0.999*
Ventriculitis, no. (%), n=369	13 (3.5)	5 (2.0)	8 (6.7)	0.032*
Pneumonia, no. (%)	58 (14.0)	23 (8.2)	35 (26.3)	<0.001
Urinary tract infection, no. (%)	9 (2.2)	5 (1.8)	4 (3.0)	0.476*
**Clinical time course**	n=415	n=282	n=133	
Ictus to hospital arrival (hour), no. (%), n=408				0.089
≤24 hours	212 (52.0)	134 (48.6)	78 (59.1)	
>24 - 72 hours	188 (46.0)	135 (48.9)	53 (40.1)	
>72 hours	8 (2.0)	7 (2.5)	1 (0.8)	
Length of hospitalization (days), mean (SD)	10.14 (9.85)	11.22 (9.5)	7.84 (10.22)	<0.001**
**Clinical outcomes**	n=415	n=282	n=133	
Hospital discharge, no. (%)	119 (28.7)	116 (41.1)	3 (2.3)	<0.001
Transferred to another hospital, no. (%)	252 (60.7)	166 (58.9)	86 (64.7)	0.259
Discharged to die, no. (%)	33 (8.0)	1 (0.4)	32 (24.1)	<0.001
Deaths:				
Died in hospital, no. (%)	71 (17.1)	0 (0.0)	71 (53.4)	<0.001
Died within 30 days of ictus, no. (%)	89 (21.4)	0 (0.0)	89 (66.9)	<0.001
Died within 90 days of ictus, no. (%)	97 (23.4)	0 (0.0)	97 (72.9)	<0.001
Neurological function:				
mRS score at hospital	1.0 (1.0-5.0)	1.0 (1.0-1.0)	5.0 (5.0-5.0)	<0.001**

discharge, median (IQR)				
mRS at hospital discharge, no. (%)				<0.001
Good (mRS of 0 to 3)	266 (64.1)	262 (92.9)	4 (3.0)	
Poor (mRS of 4 to 6)	149 (35.9)	20 (7.1)	129 (97.0)	
mRS score at 30 days of ictus, median (IQR)	2.15 (2.54)	0.51 (0.92)	5.63 (0.68)	<0.001**
mRS at 30 days of ictus, no. (%)				<0.001
Good (mRS of 0 to 3)	277 (66.7)	277 (98.2)	0 (0.0)	
Poor (mRS of 4 to 6)	138 (33.3)	5 (1.8)	133 (100)	

[a] Comparison between mRS of 0 to 3 and mRS of 4 to 6 using Chi-squared test; *Fisher's exact test; **Mann–Whitney U test.

[b] Surgical haematoma evacuation was defined as any surgical procedure evacuating epidural, subdural, intraventricular, or intraparenchymal haematoma, such as decompressive craniotomy, open craniotomy, or minimally invasive surgery.

Abbreviations: **DCI**: delayed cerebral ischemia; **EVD**: external ventricular drainage; **IQR**: interquartile range; **IVF**: intraventricular fibrinolysis; **mRS**: modified Rankin Scale; **no.**: number; **SD**: standard deviation.

Table S10. Summary table of various diagnostic indices and AUROC of the PAASH, WFNS, and H&H scales for predicting the poor outcome (mRS of 4 to 6) after ictus in patients with aneurysmal SAH

Variable	Cut-off value	Youden's index J	Sensitivity (%)	Specificity (%)	LR+	LR–	AUROC (95% CI)	p-value
**Poor outcome on day 30th after ictus**								
PAASH	≥2.5	0.558	67.4	88.4	5.834	0.369	0.840 (0.796-0.883)	<0.001
WFNS	≥3.5	0.591	75.4	83.8	4.639	0.294	0.836 (0.793-0.880)	<0.001
H&H	≥3.5	0.569	71.7	85.2	4.847	0.332	0.839 (0.795-0.883)	<0.001
**Poor outcome on day 90th after ictus**								
PAASH	≥2.5	0.553	67.7	87.6	5.452	0.397	0.838 (0.794-0.882)	<0.001
WFNS	≥3.5	0.589	75.9	83.0	4.461	0.289	0.837 (0.793-0.881)	<0.001
H&H	≥3.5	0.566	72.2	84.4	4.626	0.329	0.836 (0.791-0.881)	<0.001

Abbreviations: **AUC**, the area under the curve; **AUROC**, the area under the receiver operating characteristic; **CI**, confidence interval; **H&H**, Hunt and Hess scale; **LR**, likelihood ratio; **PAASH**, Prognosis on Admission of Aneurysmal Subarachnoid Haemorrhage; **SAH**, subarachnoid haemorrhage; **WFNS**, World Federation of Neurosurgical Societies scale.

Table S11. Factors associated with poor outcome (mRS of 4 to 6) on day 30th after ictus in patients with aneurysmal subarachnoid haemorrhage (the exposure variable was defined as the originally-suggested 5-category PAASH grading scale)

Factors	Univariable logistic regression analyses[a]				Multivariable logistic regression analyses[b]			
	OR	95% CI for OR		p-value	AOR	95% CI for AOR		p-value
		Lower	Upper			Lower	Upper	
**Demographics**								
Age ≥ 60 years	2.407	1.586	3.654	<0.001	3.104	1.347	7.153	0.008
**Risk factors of aneurysmal SAH**								
Hypertension	2.056	1.354	3.122	0.001	NA	NA	NA	NA
**Comorbidities**								

Diabetes mellitus	3.751	1.669	8.433	0.001	NA	NA	NA	NA
**Neuroimaging findings on admission**								
Location of blood within the subarachnoid space:								
Basal cistern	2.685	1.728	4.173	<0.001	NA	NA	NA	NA
Sylvian fissure	2.981	1.125	7.900	0.028	NA	NA	NA	NA
Interhemispheric fissure	1.960	1.207	3.183	0.007	NA	NA	NA	NA
Interpeduncular fossa	3.254	1.995	5.308	<0.001	12.625	3.668	43.457	<0.001
Suprasellar cistern	1.929	1.221	3.047	0.005	NA	NA	NA	NA
Ambient cistern	3.054	1.902	4.906	<0.001	NA	NA	NA	NA
Quadrigeminal cistern	5.141	3.270	8.085	<0.001	NA	NA	NA	NA
IVH	3.013	1.847	4.914	<0.001	NA	NA	NA	NA
ICH	1.860	1.142	3.029	0.013	NA	NA	NA	NA
Aneurysm locations								
PCoA aneurysm	0.554	0.299	1.026	0.061	NA	NA	NA	NA
VA aneurysm	3.341	1.265	8.820	0.015	NA	NA	NA	NA
**Severity of aneurysmal SAH on admission**								
PAASH grading scale								
I	Reference			<0.001	Reference			<0.001
II	4.219	2.182	8.158	<0.001	2.954	0.944	9.244	0.063
III	14.837	7.185	30.641	<0.001	12.625	3.668	43.457	<0.001
IV	51.605	22.630	117.682	<0.001	28.875	7.501	111.160	<0.001
V	77.895	9.240	656.657	<0.001	39.828	1.947	814.842	0.017
**Aneurysm repairs and other treatments**								

Aneurysm repairs:								
No aneurysm repair	Reference			<0.001	Reference			<0.001
Endovascular coiling	0.009	0.003	0.027	<0.001	0.012	0.003	0.048	<0.001
Surgical clipping	0.020	0.007	0.059	<0.001	0.020	0.005	0.079	<0.001
Nimodipine	0.076	0.028	0202	<0.001	NA	NA	NA	NA
**Complications**								
Rebleeding	7.868	2.537	24.396	<0.001	24.322	4.584	129.047	<0.001
DCI	7.316	2.848	18.792	<0.001	21.449	5.553	82.858	<0.001
Acute hydrocephalus	3.134	2.034	4.830	<0.001	2.419	1.031	5.679	0.042
Pneumonia	4.091	2.295	7.292	<0.001	3.261	1.251	8.505	0.016
Constant					0.375			0.232

[a]Each variable of the demographics, risk factors for aneurysmal SAH, comorbidities, initial clinical, neuroimaging and laboratory characteristics, the severity of aneurysmal SAH (i.e., PAASH scale) on admission, treatments, and complications was analysed in the univariable logistic regression model and was considered in the multivariable logistic regression model if the P-value was <0.05 in univariable logistic regression analysis, as well as clinically crucial factors.

[b]All selected variables were included in the multivariable logistic regression model with the stepwise backward elimination method. Variables, then, were deleted stepwise from the full model until all remaining variables were independently associated with poor outcomes.

Abbreviations: **AOR**, adjusted odds ratio; **CI**, confidence interval; **DCI**, delayed cerebral ischemia; **ICH**, intracerebral haemorrhage; **IVH**, intraventricular haemorrhage; **mRS**, modified Rankin Scale; **NA**, not available; **OR**, odds ratio; **PAASH**: Prognosis on Admission of Aneurysmal Subarachnoid Haemorrhage; **PCoA**, posterior communicating artery; **SAH**, subarachnoid haemorrhage; **VA**, vertebral artery.

Table S12. Factors associated with poor outcome (mRS of 4 to 6) on day 30th days ictus in patients with aneurysmal subarachnoid haemorrhage (the exposure variable was defined as the originally-suggested 5-category WFNS grading scale)

Factors	Univariable logistic regression analyses[a]				Multivariable logistic regression analyses[b]			
	OR	95% CI for OR		p-value	AOR	95% CI for AOR		p-value
		Lower	Upper			Lower	Upper	
**Demographics**								
Age ≥ 60 years	2.407	1.586	3.654	<0.001	2.581	1.159	5.747	0.020

**Risk factors of aneurysmal SAH**								
Hypertension	2.056	1.354	3.122	0.001	NA	NA	NA	NA
**Comorbidities**								
Diabetes mellitus	3.751	1.669	8.433	0.001	NA	NA	NA	NA
**Neuroimaging findings on admission**								
Location of blood within the subarachnoid space:								
Basal cistern	2.685	1.728	4.173	<0.001	NA	NA	NA	NA
Sylvian fissure	2.981	1.125	7.900	0.028	NA	NA	NA	NA
Interhemispheric fissure	1.960	1.207	3.183	0.007	NA	NA	NA	NA
Interpeduncular fossa	3.254	1.995	5.308	<0.001	2.399	1.012	5.690	0.047
Suprasellar cistern	1.929	1.221	3.047	0.005	NA	NA	NA	NA
Ambient cistern	3.054	1.902	4.906	<0.001	NA	NA	NA	NA
Quadrigeminal cistern	5.141	3.270	8.085	<0.001	NA	NA	NA	NA
IVH	3.013	1.847	4.914	<0.001	NA	NA	NA	NA
ICH	1.860	1.142	3.029	0.013	NA	NA	NA	NA
Aneurysm locations								
PCoA aneurysm	0.554	0.299	1.026	0.061	NA	NA	NA	NA
VA aneurysm	3.341	1.265	8.820	0.015	NA	NA	NA	NA
**Severity of aneurysmal SAH on admission**								
WFNS grading scale								
I	Reference			<0.001	Reference			<0.001
II	2.895	1.272	6.587	0.011	2.546	0.611	10.617	0.200
III	3.895	1.114	13.621	0.033	5.783	0.865	38.655	0.070

IV	15.630	8.391	29.117	<0.001	9.188	3.153	26.779	<0.001
V	59.812	23.648	151.281	<0.001	20.962	5.004	87.813	<0.001
**Aneurysm repairs and other treatments**								
Aneurysm repairs:								
No aneurysm repair	Reference			<0.001	Reference			<0.001
Endovascular coiling	0.009	0.003	0.027	<0.001	0.012	0.003	0.048	<0.001
Surgical clipping	0.020	0.007	0.059	<0.001	0.021	0.005	0.078	<0.001
Nimodipine	0.076	0.028	0202	<0.001	NA	NA	NA	NA
**Complications**								
Rebleeding	7.868	2.537	24.396	<0.001	20.247	3.821	107.282	<0.001
DCI	7.316	2.848	18.792	<0.001	17.436	4.505	67.472	<0.001
Acute hydrocephalus	3.134	2.034	4.830	<0.001	2.331	1.029	5.281	0.042
Pneumonia	4.091	2.295	7.292	<0.001	3.588	1.418	9.077	0.007
Constant					0.461			0.335

[a]Each variable of the demographics, risk factors for aneurysmal SAH, comorbidities, initial clinical, neuroimaging and laboratory characteristics, the severity of aneurysmal SAH (i.e., WFNS scale) on admission, treatments, and complications was analysed in the univariable logistic regression model and was considered in the multivariable logistic regression model if the P-value was <0.05 in univariable logistic regression analysis, as well as clinically crucial factors.

[b]All selected variables were included in the multivariable logistic regression model with the stepwise backward elimination method. Variables, then, were deleted stepwise from the full model until all remaining variables were independently associated with poor outcomes.

Abbreviations: **AOR**, adjusted odds ratio; **CI**, confidence interval; **DCI**, delayed cerebral ischemia; **ICH**, intracerebral haemorrhage; **IVH**, intraventricular haemorrhage; **mRS**, modified Rankin Scale; **NA**, not available; **OR**, odds ratio; **PCoA**, posterior communicating artery; **SAH**, subarachnoid haemorrhage; **VA**, vertebral artery; **WFNS**, World Federation of Neurosurgical Societies.

Table S13. Factors associated with poor outcome (mRS of 4 to 6) on day 30th after ictus in patients with aneurysmal subarachnoid haemorrhage (the exposure variable was defined as the originally-suggested 5-category H&H grading scale)

Factors	Univariable logistic regression analyses[a]				Multivariable logistic regression analyses[b]			
	OR	95% CI for OR		p-value	AOR	95% CI for AOR		p-value
		Lower	Upper			Lower	Upper	
**Demographics**								
Age ≥ 60 years	2.407	1.586	3.654	<0.001	2.769	1.192	6.429	0.018
**Risk factors of aneurysmal SAH**								
Hypertension	2.056	1.354	3.122	0.001	NA	NA	NA	NA
**Comorbidities**								
Diabetes mellitus	3.751	1.669	8.433	0.001	NA	NA	NA	NA
**Neuroimaging findings on admission**								
Location of blood within the subarachnoid space:								
Basal cistern	2.685	1.728	4.173	<0.001	NA	NA	NA	NA
Sylvian fissure	2.981	1.125	7.900	0.028	NA	NA	NA	NA
Interhemispheric fissure	1.960	1.207	3.183	0.007	NA	NA	NA	NA
Interpeduncular fossa	3.254	1.995	5.308	<0.001	2.259	0.926	5.508	0.073
Suprasellar cistern	1.929	1.221	3.047	0.005	NA	NA	NA	NA
Ambient cistern	3.054	1.902	4.906	<0.001	NA	NA	NA	NA
Quadrigeminal cistern	5.141	3.270	8.085	<0.001	NA	NA	NA	NA
IVH	3.013	1.847	4.914	<0.001	NA	NA	NA	NA
ICH	1.860	1.142	3.029	0.013	NA	NA	NA	NA
Aneurysm locations								
PCoA aneurysm	0.554	0.299	1.026	0.061	NA	NA	NA	NA

VA aneurysm	3.341	1.265	8.820	0.015	NA	NA	NA	NA
**Severity of aneurysmal SAH on admission**								
H&H grading scale								
I	Reference			<0.001	Reference			<0.001
II	0.784	0.269	2.287	0.656	4.454	0.708	28.005	0.111
III	3.535	1.206	10.358	0.021	12.630	1.840	86.713	0.010
IV	7.360	2.478	21.860	<0.001	20.156	2.951	137.679	0.002
V	38.000	12.973	111.306	<0.001	69.898	10.687	457.156	<0.001
**Aneurysm repairs and other treatments**								
Aneurysm repairs:								
No aneurysm repair	Reference			<0.001	Reference			<0.001
Endovascular coiling	0.009	0.003	0.027	<0.001	0.009	0.002	0.038	<0.001
Surgical clipping	0.020	0.007	0.059	<0.001	0.014	0.003	0.058	<0.001
Nimodipine	0.076	0.028	0202	<0.001				
**Complications**								
Rebleeding	7.868	2.537	24.396	<0.001	22.100	4.130	118.265	<0.001
DCI	7.316	2.848	18.792	<0.001	32.198	6.512	159.197	<0.001
Acute hydrocephalus	3.134	2.034	4.830	<0.001	2.381	1.035	5.479	0.041
Pneumonia	4.091	2.295	7.292	<0.001	3.968	1.531	10.285	0.005
Constant					0.184			0.097

[a]Each variable of the demographics, risk factors for aneurysmal SAH, comorbidities, initial clinical, neuroimaging and laboratory characteristics, the severity of aneurysmal SAH (i.e., H&H scale) on admission, treatments, and complications was analysed in the univariable logistic regression model and was considered in the multivariable logistic regression model if the P-value was <0.05 in univariable logistic regression analysis, as well as clinically crucial factors.

[b]All selected variables were included in the multivariable logistic regression model with the stepwise backward elimination method. Variables, then, were deleted stepwise from the full model until all remaining variables were independently associated with poor outcomes.

Abbreviations: **AOR**, adjusted odds ratio; **CI**, confidence interval; **DCI**, delayed cerebral ischemia; **H&H**, Hunt and Hess; **ICH**, intracerebral haemorrhage; **IVH**,

intraventricular haemorrhage; **mRS**, modified Rankin Scale; **NA**, not available; **OR**, odds ratio; **PCoA**, posterior communicating artery; **SAH**, subarachnoid haemorrhage; **VA**, vertebral artery.

Table 14. Factors associated with poor outcome (mRS of 4 to 6) on day 90th after ictus in patients with aneurysmal subarachnoid haemorrhage (the exposure variable was defined as the originally-suggested 5-category PAASH grading scale)

Factors	Univariable logistic regression analyses[a]				Multivariable logistic regression analyses[b]			
	OR	95% CI for OR		p-value	AOR	95% CI for AOR		p-value
		Lower	Upper			Lower	Upper	
**Demographics**								
Age ≥ 60 years	2.581	1.691	3.939	<0.001	3.613	0.970	9.977	0.056
**Risk factors of aneurysmal SAH**								
Hypertension	2.133	1.400	3.250	<0.001	NA	NA	NA	NA
**Comorbidities**								
Diabetes mellitus	3.986	1.772	8.968	0.001	NA	NA	NA	NA
**Neuroimaging findings on admission**								
Location of blood within the subarachnoid space:								
Basal cistern	2.718	1.738	4.251	<0.001	NA	NA	NA	NA
Sylvian fissure	2.811	1.060	7.454	0.038	NA	NA	NA	NA
Interhemispheric fissure	2.063	1.257	3.385	0.004	NA	NA	NA	NA
Interpeduncular fossa	3.206	1.953	5.264	<0.001	NA	NA	NA	NA
Suprasellar cistern	1.882	1.186	2.986	0.007	NA	NA	NA	NA
Ambient cistern	2.990	1.852	4.829	<0.001	NA	NA	NA	NA
Quadrigeminal cistern	4.414	3.431	8.545	<0.001	2.317	0.982	5.467	0.055

IVH	2.793	1.711	4.559	<0.001	NA	NA	NA	NA
ICH	1.879	1.151	3.068	0.012	NA	NA	NA	NA
Aneurysm locations								
PCoA aneurysm	0.590	0.318	1.094	0.094	NA	NA	NA	NA
VA aneurysm	3.542	1.341	9.356	0.011	NA	NA	NA	NA
**Severity of aneurysmal SAH on admission**								
PAASH grading scale								
I	Reference			<0.001	Reference			<0.001
II	4.235	2.164	8.287	<0.001	3.112	0.970	9.977	0.056
III	13.478	6.512	27.896	<0.001	10.120	2.874	35.629	<0.001
IV	48.848	21.716	109.879	<0.001	24.649	6.358	95.559	<0.001
V	82.667	9.781	698.704	<0.001	35.714	1.836	694.820	0.018
**Aneurysm repairs and other treatments**								
Aneurysm repairs:								
No aneurysm repair	Reference			<0.001	Reference			<0.001
Endovascular coiling	0.011	0.004	0.030	<0.001	0.014	0.004	0.053	<0.001
Surgical clipping	0.023	0.009	0.062	<0.001	0.028	0.008	0.101	<0.001
Nimodipine	0.071	0.027	0.191	<0.001	NA	NA	NA	NA
**Complications**								
Rebleeding	6.133	2.138	17.594	0.001	28.247	5.244	152.148	<0.001
DCI	6.245	2.538	15.365	<0.001	17.236	4.392	67.640	<0.001
Acute hydrocephalus	3.134	2.028	4.842	<0.001	2.552	1.080	6.032	0.033
Pneumonia	4.022	2.263	7.148	<0.001	4.133	1.578	10.825	0.004
Constant					0.351			0.165

[a]Each variable of the demographics, risk factors for aneurysmal SAH, comorbidities, initial clinical, neuroimaging and laboratory characteristics, the severity of aneurysmal

SAH (i.e., PAASH scale) on admission, treatments, and complications was analysed in the univariable logistic regression model and was considered in the multivariable logistic regression model if the P-value was <0.05 in univariable logistic regression analysis, as well as clinically crucial factors.

[b]All selected variables were included in the multivariable logistic regression model with the stepwise backward elimination method. Variables, then, were deleted stepwise from the full model until all remaining variables were independently associated with poor outcomes.

Abbreviations: **AOR**, adjusted odds ratio; **CI**, confidence interval; **DCI**, delayed cerebral ischemia; **ICH**, intracerebral haemorrhage; **IVH**, intraventricular haemorrhage; **mRS**, modified Rankin Scale; **NA**, not available; **OR**, odds ratio; **PAASH**: Prognosis on Admission of Aneurysmal Subarachnoid Haemorrhage; **PCoA**, posterior communicating artery; **SAH**, subarachnoid haemorrhage; **VA**, vertebral artery.

Table S15. Factors associated with poor outcome (mRS of 4 to 6) on day 90th after ictus in patients with aneurysmal subarachnoid haemorrhage (the exposure variable was defined as the originally-suggested 5-category WFNS grading scale)

Factors	Univariable logistic regression analyses[a]				Multivariable logistic regression analyses[b]			
	OR	95% CI for OR		p-value	AOR	95% CI for AOR		p-value
		Lower	Upper			Lower	Upper	
**Demographics**								
Age ≥ 60 years	2.581	1.691	3.939	<0.001	3.124	1.380	7.073	0.006
**Risk factors of aneurysmal SAH**								
Hypertension	2.133	1.400	3.250	<0.001	NA	NA	NA	NA
**Comorbidities**								
Diabetes mellitus	3.986	1.772	8.968	0.001	NA	NA	NA	NA
**Neuroimaging findings on admission**								
Location of blood within the subarachnoid space:								
Basal cistern	2.718	1.738	4.251	<0.001	NA	NA	NA	NA
Sylvian fissure	2.811	1.060	7.454	0.038	NA	NA	NA	NA
Interhemispheric fissure	2.063	1.257	3.385	0.004	NA	NA	NA	NA

Interpeduncular fossa	3.206	1.953	5.264	<0.001	NA	NA	NA	NA
Suprasellar cistern	1.882	1.186	2.986	0.007	NA	NA	NA	NA
Ambient cistern	2.990	1.852	4.829	<0.001	NA	NA	NA	NA
Quadrigeminal cistern	4.414	3.431	8.545	<0.001	2.178	0.923	5.142	0.076
IVH	2.793	1.711	4.559	<0.001	NA	NA	NA	NA
ICH	1.879	1.151	3.068	0.012	NA	NA	NA	NA
Aneurysm locations								
PCoA aneurysm	0.590	0.318	1.094	0.094	NA	NA	NA	NA
VA aneurysm	3.542	1.341	9.356	0.011	NA	NA	NA	NA
**Severity of aneurysmal SAH on admission**								
WFNS grading scale								
I	Reference			<0.001	Reference			<0.001
II	2.719	1.164	6.350	0.021	2.725	0.635	11.686	0.177
III	4.133	1.177	14.520	0.027	4.813	0.691	33.541	0.113
IV	14.618	7.803	27.383	<0.001	7.943	2.678	23.558	<0.001
V	63.476	24.947	161.511	<0.001	20.823	4.842	89.556	<0.001
**Aneurysm repairs and other treatments**								
Aneurysm repairs:								
No aneurysm repair	Reference			<0.001	Reference			<0.001
Endovascular coiling	0.011	0.004	0.030	<0.001	0.014	0.004	0.050	<0.001
Surgical clipping	0.023	0.009	0.062	<0.001	0.027	0.008	0.096	<0.001
Nimodipine	0.071	0.027	0.191	<0.001	NA	NA	NA	NA
**Complications**								
Rebleeding	6.133	2.138	17.594	0.001	24.221	4.507	130.161	<0.001

DCI	6.245	2.538	15.365	<0.001	13.816	3.641	52.427	<0.001
Acute hydrocephalus	3.134	2.028	4.842	<0.001	2.451	1.073	5.601	0.033
Pneumonia	4.022	2.263	7.148	<0.001	4.454	1.729	11.479	0.002
Constant					0.438			0.262

[a]Each variable of the demographics, risk factors for aneurysmal SAH, comorbidities, initial clinical, neuroimaging and laboratory characteristics, the severity of aneurysmal SAH (i.e., WFNS scale) on admission, treatments, and complications was analysed in the univariable logistic regression model and was considered in the multivariable logistic regression model if the P-value was <0.05 in univariable logistic regression analysis, as well as clinically crucial factors.

[b]All selected variables were included in the multivariable logistic regression model with the stepwise backward elimination method. Variables, then, were deleted stepwise from the full model until all remaining variables were independently associated with poor outcomes.

Abbreviations: **AOR**, adjusted odds ratio; **CI**, confidence interval; **DCI**, delayed cerebral ischemia; **ICH**, intracerebral haemorrhage; **IVH**, intraventricular haemorrhage; **mRS**, modified Rankin Scale; **NA**, not available; **OR**, odds ratio; **PCoA**, posterior communicating artery; **SAH**, subarachnoid haemorrhage; **VA**, vertebral artery; **WFNS**, World Federation of Neurosurgical Societies.

Table S16. Factors associated with poor outcome (mRS of 4 to 6) on day 90th after ictus in patients with aneurysmal subarachnoid haemorrhage (the exposure variable was defined as the originally-suggested 5-category H&H grading scale)

Factors	Univariable logistic regression analyses[a]				Multivariable logistic regression analyses[b]			
	OR	95% CI for OR		p-value	AOR	95% CI for AOR		p-value
		Lower	Upper			Lower	Upper	
**Demographics**								
Age ≥ 60 years	2.581	1.691	3.939	<0.001	3.391	1.435	8.017	0.005
**Risk factors of aneurysmal SAH**								
Hypertension	2.133	1.400	3.250	<0.001	NA	NA	NA	NA
**Comorbidities**								
Diabetes mellitus	3.986	1.772	8.968	0.001	NA	NA	NA	NA
**Neuroimaging findings on admission**								

Location of blood within the subarachnoid space:								
Basal cistern	2.718	1.738	4.251	<0.001	NA	NA	NA	NA
Sylvian fissure	2.811	1.060	7.454	0.038	NA	NA	NA	NA
Interhemispheric fissure	2.063	1.257	3.385	0.004	NA	NA	NA	NA
Interpeduncular fossa	3.206	1.953	5.264	<0.001	NA	NA	NA	NA
Suprasellar cistern	1.882	1.186	2.986	0.007	NA	NA	NA	NA
Ambient cistern	2.990	1.852	4.829	<0.001	NA	NA	NA	NA
Quadrigeminal cistern	4.414	3.431	8.545	<0.001	2.202	0.920	5.272	0.076
IVH	2.793	1.711	4.559	<0.001	NA	NA	NA	NA
ICH	1.879	1.151	3.068	0.012	NA	NA	NA	NA
Aneurysm locations								
PCoA aneurysm	0.590	0.318	1.094	0.094	NA	NA	NA	NA
VA aneurysm	3.542	1.341	9.356	0.011	NA	NA	NA	NA
**Severity of aneurysmal SAH on admission**								
H&H grading scale								
I	Reference			<0.001	Reference			<0.001
II	0.727	0.247	2.139	0.563	3.596	0.596	21.685	0.163
III	3.273	1.112	9.631	0.031	10.456	1.624	67.312	0.013
IV	6.769	2.277	20.121	0.001	13.888	2.196	87.811	0.005
V	32.889	11.362	95.201	<0.001	49.363	7.977	305.477	<0.001
**Aneurysm repairs and other treatments**								
Aneurysm repairs:								
No aneurysm repair	Reference			<0.001	Reference			<0.001
Endovascular coiling	0.011	0.004	0.030	<0.001	0.010	0.003	0.041	<0.001

Surgical clipping	0.023	0.009	0.062	<0.001	0.019	0.005	0.072	<0.001
Nimodipine	0.071	0.027	0.191	<0.001	NA	NA	NA	NA
**Complications**								
Rebleeding	6.133	2.138	17.594	0.001	27.015	5.136	142.091	<0.001
DCI	6.245	2.538	15.365	<0.001	23.161	5.119	104.788	<0.001
Acute hydrocephalus	3.134	2.028	4.842	<0.001	2.404	1.049	5.509	0.038
Pneumonia	4.022	2.263	7.148	<0.001	4.971	1.897	13.024	0.001
Constant					0.199			0.092

[a]Each variable of the demographics, risk factors for aneurysmal SAH, comorbidities, initial clinical, neuroimaging and laboratory characteristics, the severity of aneurysmal SAH (i.e., H&H scale) on admission, treatments, and complications was analysed in the univariable logistic regression model and was considered in the multivariable logistic regression model if the P-value was <0.05 in univariable logistic regression analysis, as well as clinically crucial factors.

[b]All selected variables were included in the multivariable logistic regression model with the stepwise backward elimination method. Variables, then, were deleted stepwise from the full model until all remaining variables were independently associated with poor outcomes.

Abbreviations: **AOR**, adjusted odds ratio; **CI**, confidence interval; **DCI**, delayed cerebral ischemia; **H&H**, Hunt and Hess; **ICH**, intracerebral haemorrhage; **IVH**, intraventricular haemorrhage; **mRS**, modified Rankin Scale; **NA**, not available; **OR**, odds ratio; **PCoA**, posterior communicating artery; **SAH**, subarachnoid haemorrhage; **VA**, vertebral artery.

Table S17. Breakdown of missing data

Variables	Number of patients with missing data
Prehospital setting	
Transferred from local hospitals	0
Hospital taken to	
Viet Duc	0
Bach Mai	0
Hanoi Medical University	0
**Demographics**	
Age (year)	0
Gender (male)	0
**Risk factors of aneurysmal SAH**	
Cigarette smoking	0
Hypertension	2
Genetic risk	0
Alcohol consumption	14
Sympathomimetic drugs	2
Estrogen deficiency	4
Antithrombotic therapy	0
Elevated total cholesterol	0
**Comorbidities**	
Cerebrovascular disease	0
Chronic cardiac failure	0
Coronary artery disease/MI	0
COPD/Asthma	0
Active neoplasm	0
Chronic renal failure	0
Ulcer disease	0
Diabetes mellitus	0
Haematological disease	0
**Onset symptoms**	
Sudden-onset, severe headache	0
Vomiting	0
Neck pain or stiffness	0
Photophobia	0
Blurred or double vision	0
Brief loss of consciousness	0
Seizures	0
**Clinical presentation on admission**	
GCS score	0
Focal neurological deficits	0

**Neuroimaging findings on admission**	
Location of blood within the subarachnoid space	
Basal cistern	4
Sylvian fissure	4
Interhemispheric fissure	2
Interpeduncular fossa	3
Suprasellar cistern	3
Ambient cistern	3
Quadrigeminal cistern	3
IVH	0
ICH	0
Subdural haemorrhage	0
Hydrocephalus	0
Evans' index	23
Hypodense lesions on computed tomography	2
**Admission laboratory investigations**	
Platelets (G/L)	7
PT-INR	17
**Initial severity of aneurysmal SAH**	
PAASH score	0
WFNS score	0
H&H score	0
Fisher score	1
**Aneurysm repairs and other treatments**	
No aneurysm repair	0
Endovascular coiling	0
Surgical clipping	0
Surgical hematoma evacuation or decompressive craniotomy	0
EVD	1
IVF	0
Nimodipine	52
**Complications**	
Late rebleeding	4
DCI	6
Acute hydrocephalus	0
Hyponatremia	0
Seizures	0
Chronic hydrocephalus	71
Ventriculitis	46
Pneumonia	0

Urinary tract infection	0
**Clinical time course**	
Ictus to hospital arrival (hour)	0
Length of hospitalization (days)	0
**Clinical outcomes**	
Hospital discharge	0
Transferred to another hospital	0
Discharged to die	0
Deaths:	
Died in hospital	0
Died within 30 days of ictus	0
Died within 90 days of ictus	0
Neurological function:	
mRS score at hospital discharge	0
mRS score at 30 days of ictus	0
mRS at 90 days of ictus	0

Abbreviations

COPD: chronic obstructive pulmonary disease; **DCI**: delayed cerebral ischemia; **EVD**: external ventricular drainage; **GCS**: Glasgow Coma Scale; **H&H**: Hunt and Hess; **ICH**: intracerebral haemorrhage; **IVF**: intraventricular fibrinolysis; **IVH**: intraventricular haemorrhage; **MI**: myocardial ischemia; **PAASH**: Prognosis on Admission of Aneurysmal Subarachnoid Haemorrhage; **PT-INR**: prothrombin time with international normalized ratio; **SAH**: subarachnoid haemorrhage; **WFNS**: World Federation of Neurological Surgeons.
